# Plant‐Mediated Fabrication of Silver Nanoparticles Using *Elaeagnus angustifolia* Flowers: In Vitro Evaluation of Antibacterial, Antifungal, Antioxidant, and Toxic Effects and Biomolecular Interactions With ct‐DNA and HSA

**DOI:** 10.1155/bca/9779231

**Published:** 2026-06-14

**Authors:** Nahid Shahabadi, Kani Shalmashi, Sedigheh Shokraei, Leila Soltani

**Affiliations:** ^1^ Department of Inorganic Chemistry, Faculty of Chemistry, Razi University, Kermanshah, Iran, razi.ac.ir; ^2^ Department of Animal Sciences, College of Agriculture and Natural Resources, Razi University, Kermanshah, Iran, razi.ac.ir

**Keywords:** antibacterial activity, antioxidant activity, cytotoxicity, Elaeagnus angustifolia flowers, silver nanoparticle–ct-DNA interaction, silver nanoparticle–protein interaction, silver nanoparticles by green synthesis

## Abstract

This study aimed to biosynthesize silver nanoparticles using *Elaeagnus angustifolia* flower *buds*—an Iranian medicinal plant extract rich in bioactive phytochemicals and evaluate their antimicrobial potential through complementary broth microdilution (MIC/MBC) and agar well diffusion assays, alongside an assessment of their anticancer and biomolecular interaction properties. The synthesis leverages natural reducing and capping agents (phenols, flavonoids, tannins) to produce stable, spherical AgNPs (FEA@AgNPs), characterized by UV–vis (*λ*_max_ = 439 nm), XRD (∼10 nm, face‐centered cubic), FESEM, TEM (6.61 nm), and FT‐IR. A zeta potential of −32.68 mV confirmed colloidal stability. The nanoparticles were stable for over a month, indicating that *E. angustifolia* flower aqueous extract is suitable for their preparation and stabilization. FEA@AgNPs showed moderate activity against *S. aureus* (12 vs. 24 mm for gentamicin) and no agar diffusion inhibition against *E. coli*, despite a MIC of 37.5 μg/mL in the broth assay. The antioxidant results show 55% DPPH radical scavenging at 160 μg/mL. Notably, they induced dose‐ and time‐dependent cytotoxicity in PC3 and AGS cancer cells, with IC_50_ values of 7.49 and 5.33 μg/mL, respectively, after 72 h (*p* < 0.05). Spectroscopic analyses revealed a strong binding affinity to calf thymus DNA and human serum albumin, suggesting biomolecular interaction capacity relevant to drug delivery. This work provides a green, efficient route to multifunctional AgNPs, bridging traditional herbal knowledge and bioinorganic nanomedicine for potential applications in infection control and oncology.

## 1. Introduction

Silver nanoparticles (AgNPs) are among the most promising nanomaterials in biomedicine, valued for their antimicrobial, anticancer, and antioxidant activities. While conventional synthesis methods rely on toxic chemicals, green synthesis using plant extracts offers a sustainable alternative—leveraging natural phytochemicals as dual reducing and capping agents to produce biocompatible, functionalized nanoparticles [1–4].

The choice of botanical source has a critical influence on the stability, morphology, and bioactivity of biosynthesized AgNPs [2, 5]. In recent years, plant‐derived antioxidants have emerged as promising prophylactic and therapeutic agents in the management of oxidative stress‐related diseases. Some researchers suggest a paradigm shift toward natural, multitargeted approaches in healthcare. *Elaeagnus angustifolia,* a species native to parts of Europe and Asia—including Iran—and belonging to the Elaeagnaceae family, has been widely used in traditional medicine across Western Asia—Iran—for its diverse therapeutic properties. Various parts of the plant—particularly, the flowers and fruits—have been traditionally employed to treat nausea, vomiting, jaundice, asthma, abdominal distension, and tetanus, while the fruit is used as an analgesic in rheumatoid arthritis [1]. Scientific investigations have validated several of these applications, demonstrating that E. angustifolia possesses anti‐inflammatory, antiulcerogenic, and relaxant effects on muscle [1, 6].

Crucially, the flowers of *E. angustifolia* are rich in redox‐active phytochemicals, including phenolic compounds and flavonoids, which exhibit potent antioxidant activity by neutralizing reactive oxygen species (ROS) and protecting cellular components from oxidative damage [1, 7]. This antioxidant efficacy has been proposed for pharmaceutical applications, as natural food supplements and as stabilizers against oxidative deterioration in food systems [7]. Given the well‐documented role of oxidative stress in aging and chronic diseases—including carcinogenesis—the bioactive constituents of *E. angustifolia* suggest significant therapeutic potential.

Given this compelling combination of high phenolic content, validated antioxidant power, and documented bioactivities, the flower extract of *E. angustifolia* was selected in the present study as a green, sustainable candidate for comprehensive evaluation of its antimicrobial, anticancer, and antioxidant properties. This approach aligns with the growing demand for multifunctional, plant‐based agents that can simultaneously target microbial infection, cancer progression, and oxidative stress—three interconnected pillars of modern pathophysiology.

The synthesized AgNPs were subsequently characterized using a range of analytical techniques, including ultraviolet–visible spectroscopy (UV–vis), Fourier‐transform infrared spectroscopy (FT‐IR), energy‐dispersive x‐ray spectroscopy (EDX), XRD, field emission scanning electron microscopy (FESEM), and transmission electron microscopy (TEM). The antioxidant capacity, as well as the antibacterial and antifungal properties, of the synthesized AgNPs was evaluated against selected human pathogenic microorganisms, including *Escherichia coli, Staphylococcus aureus, and Candida albicans*, along with their binding affinity toward *calf thymus DNA (ct-DNA)* and *human serum albumin (HSA)*. Additionally, the anticancer potential of AgNPs was investigated using in vitro assays on *fibroblast, gastric cancer (*AGS*), and prostate cancer (PC3) cell lines*.

This study highlights the promising biomedical applications of plant‐derived AgNPs, particularly those synthesized using species from the Elaeagnaceae family, when combined with advances in nanotechnology. By integrating traditional ethnobotanical knowledge with nanotechnology, this work paves the way for a multifunctional, plant‐based nanotherapeutic platform.

## 2. Materials and Methods

For the bioinspired synthesis of nanoparticles, analytical‐grade silver nitrate (AgNO_3_) was used. Mueller‐Hinton agar and Mueller‐Hinton broth (MHB), employed for the evaluation of antibacterial and antifungal activities, were purchased from Merck. Bacterial and fungal strains—*E. coli*, *S. aureus*, and *C. albicans*—were obtained from the Pasteur Institute of Iran Cell Bank.

Cell culture reagents, including trypsin, penicillin, streptomycin, physiological serum, and culture medium (RPMI‐1640), were sourced from Gibco (Thermo Fisher Scientific). Human gastric adenocarcinoma cells (AGS; IBRC C10071) were provided by the Iranian Biological Resource Center (IBRC). PC3 (C427) cells and human foreskin fibroblast (HFFF2), used as normal controls, were obtained from the Pasteur Institute of Iran.

For interaction studies, Tris(hydroxymethyl)aminomethane–hydrochloride (Tris–HCl), Hoechst 33258 (HO), acridine orange (AO), HSA, and ct‐DNA were procured from Sigma‐Aldrich and Merck Millipore. The concentration of ct‐DNA was determined spectrophotometrically at 260 nm using a molar extinction coefficient (*ε*) of 6600 L mol^−1^ cm^−1^. The purity of ct‐DNA was confirmed by an *A*_260_/*A*_280_ ratio > 1.80. Stock solutions of HO and AO (1.00 × 10^−3^ M) were prepared in distilled water.

Additionally, 3‐(4,5‐dimethylthiazol‐2‐yl)‐2,5‐diphenyltetrazolium bromide (MTT) and 2,2‐diphenyl‐1‐picrylhydrazyl (DPPH) were obtained from Sigma‐Aldrich.

All chemicals and reagents were used as received, without further purification. Deionized (DI) water (conductivity: 0.2 μS/cm; resistivity: 18.2 MΩ·cm at 25°C) was used throughout all experimental procedures.

### 2.1. Preparation of the Flower of the *E. Angustifolia* Plant Extract

In this study, flowers of *E. angustifolia* (Elaeagnaceae) were collected in May 2024 from West Azerbaijan Province, Iran. The plant material, with voucher specimen No. 202 (HRU), has been deposited at the Herbarium of Razi University, Faculty of Agricultural Sciences and Engineering, University of Kermanshah. The collected flowers were meticulously cleaned by washing them with tap water, followed by DI water to remove any adhering dust or debris. Subsequently, the flowers were shade‐dried and then finely ground into a homogeneous powder using an electric blender. A 5‐g portion of this powdered plant material was accurately weighed and subjected to an extraction process. The extraction was performed using 100 mL of DI water at a temperature of 70°C for 30 min with continuous stirring. The resulting extract was then filtered through Whatman filter paper No. 1 to separate the plant material from the extracted solution. The filtered extract was stored at 4°C for future use in the subsequent experiments.

### 2.2. Green Synthesis of AgNPs

For the green synthesis of AgNPs, an aqueous flower extract of *E. angustifolia*, prepared in a prior step, was utilized. In this process, 15 mL of the plant extract was slowly added dropwise to 100 mL of 0.01 M silver nitrate solution contained in a 250‐mL round‐bottom flask, which was wrapped in aluminum foil to prevent light exposure.

The resulting mixture was magnetically stirred gently for 15 min at 45°C, following which the temperature was raised to 70°C. The reaction was subsequently maintained at this temperature for approximately 1 hour under continuous stirring. The visual observation of a color change in the solution after 30 min was noted.

The formation of AgNPs was initially observed through a visual color change from yellow to dark brown. Upon completion of the reaction, the biosynthesized nanoparticles were separated via centrifugation at 5000 rpm for 20 min. This step was repeated three times to ensure the removal of any unreacted silver ions and loosely bound materials associated with the FEA@AgNPs.

Aliquots of the nanoparticle suspension were analyzed using a double‐beam UV–vis spectrophotometer over a wavelength range of 200–600 nm to confirm nanoparticle synthesis. Subsequently, the concentrated nanoparticle solution was dried in a hot air oven at 55°C for 5 h to obtain a stable powder form.

### 2.3. Qualitative Phytochemical Analysis of the Aqueous Flower Extract of *E. Angustifolia* [2]

A comprehensive preliminary phytochemical screening was conducted to identify the presence of various bioactive constituents in the freshly prepared aqueous flower extract of E. angustifolia. The analysis targeted major secondary metabolites such as phenols, terpenoids, tannins, flavonoids, saponins, steroids, anthraquinones, and alkaloids, using well‐established standard phytochemical methods. This investigation provides valuable insights into the plant’s potential therapeutic properties and its possible applications in pharmaceutical and biomedical fields.

An initial phytochemical screening was carried out to identify the bioactive constituents present in the aqueous flower extract of E. angustifolia, following established analytical protocols.

The primary aim of this screening was to perform a qualitative evaluation of various phytochemical constituents present in the flower extract (FEA). The methodologies employed for the phytochemical analysis are summarized in Table 1.

**TABLE 1:** Qualitative phytochemical analysis of the flower of *Elaeagnus angustifolia* extractions.

(1) To detect the presence of phenolic compounds, 1 mL of the *Elaeagnus angustifolia* flower extract was treated with 2 mL of a 5% ferric chloride (FeCl_3_) solution. The formation of a dark black coloration indicated a positive result for phenols [3]
(2) To identify the presence of alkaloids, 3 mL of the *E. angustifolia* flower extract was mixed with 3 mL of 1% hydrochloric acid (HCl) and heated in a water bath for 20 min. After cooling to room temperature, 1 mL of Mayer’s reagent was added dropwise to the solution. The appearance of a greenish cream precipitate confirmed the presence of alkaloids [4]
(3) To detect flavonoids, 3 mL of the *E. angustifolia* flower extract was mixed with 1 mL of 10% sodium hydroxide (NaOH) solution. The development of an intense yellow coloration was observed, indicating a positive result for the presence of flavonoids [8]
(4) For the detection of anthraquinones, 0.2 mL of the E. angustifolia flower extract was mixed with 5 mL of chloroform and 5 mL of ammonia solution. The appearance of a bright pink color in the aqueous layer following phase separation indicated a positive result for the presence of anthraquinones [9]
(5) To detect the presence of terpenoids and steroids, 5 mL of the E. angustifolia flower extract was mixed with 2 mL of chloroform, followed by the careful addition of 3 mL of concentrated sulfuric acid. The formation of a reddish‐brown coloration at the interface indicated the presence of terpenoids, while a red color observed in the lower layer suggested the presence of steroids [8]
(6) To detect tannins, 0.5 mL of the E. angustifolia flower extract was mixed with 1 mL of distilled water and two drops of ferric chloride solution. The development of a blue‐black coloration confirmed the presence of tannins [9]
(7) To detect the presence of saponins, 0.2 mL of the E. angustifolia flower extract was shaken vigorously with 5 mL of distilled water and then heated to a boil. The formation of persistent froth or foam indicated a positive result for saponins [3]

### 2.4. Characterization Technique of AgNPs

Biosynthesized NPs are characterized by key physicochemical properties such as size, shape, morphology, dispersion, stability, composition, crystallinity, and surface functional groups. A variety of analytical techniques were employed to evaluate these characteristics, including FT‐IR, UV–visible spectroscopy, TEM, FESEM, x‐ray diffraction (XRD), energy‐dispersive x‐ray spectroscopy (EDX), zeta potential analysis for surface charge determination, and dynamic light scattering (DLS) [10].

#### 2.4.1. UV–Vis Spectroscopy

UV–visible spectroscopy serves as a key analytical tool for the indirect assessment of metal NP biosynthesis and their stability in aqueous media. This technique provides insights into the formation kinetics, size, shape, and surface plasmon resonance (SPR) characteristics of the nanoparticles. Additionally, it enables the detection of particle agglomeration or changes in size during synthesis or storage [11].

UV–visible spectral analysis was performed using a NORDANTEC double‐beam spectrophotometer (Model T80), equipped with “UV Win Lab” software for data acquisition and analysis. Baseline correction was carried out using a blank reference to ensure accurate spectral measurements. The formation of AgNPs was monitored by recording absorbance spectra within the wavelength range of 200–800 nm, using a spectral resolution of 2 nm.

#### 2.4.2. FT‐IR Spectroscopy

FT‐IR spectroscopy was employed to identify the functional groups of biomolecules responsible for the reduction and capping of biosynthesized AgNPs. The FT‐IR spectra of the aqueous flower extract of *E. angustifolia*–mediated AgNPs (FEA‐AgNPs) were recorded using a Shimadzu IR Prestige‐21 FT‐IR spectrophotometer with the KBr pellet technique. Spectra were acquired in the wavenumber range of 4000–400 cm^−1^ at a resolution of 2 cm^−1^ [12].

#### 2.4.3. Zeta Potential and DLS

Zeta potential refers to the electrostatic charge present on the surface of nanoparticles suspended in a liquid medium, and it serves as an important indicator of colloidal stability and particle dispersion behavior. This information is crucial for understanding the surface chemistry of the nanoparticles and assessing the long‐term stability of their colloidal dispersions. Measurement techniques for zeta potential include electrophoresis, streaming potential, and laser Doppler velocimetry. These methods enable the assessment of surface chemistry, facilitate predictions regarding colloidal stability, and contribute to the optimization of formulations across a variety of applications [11].

DLS is a noninvasive analytical technique widely used to determine the size distribution of nanoparticles and macromolecules in suspension. It functions by measuring the intensity fluctuations of scattered laser light caused by the Brownian motion of particles. These fluctuations are then analyzed using the Stokes–Einstein equation to calculate the hydrodynamic diameter of the particles. DLS provides critical information regarding the size, polydispersity, and diffusion behavior of colloidal systems, making it a valuable tool in nanoparticle characterization. However, its accuracy may be compromised in the presence of multiple scattering events, which can limit its effectiveness in highly concentrated or turbid samples [11].

In this study, the zeta potential and initial size distribution of the synthesized AgNPs were measured using a HORIBA SZ‐100 and a DLS Nano DS analyzer (SN 166), respectively.

#### 2.4.4. TEM

Accurately measuring nanoparticle size is essential in nanomaterial research, since size plays a key role in determining both their characteristics and potential uses. One of the most effective methods for assessing particle size is TEM, which offers both quantitative and qualitative insights through its direct imaging capabilities. Such advantages should be duly considered when employing this technique. TEM serves as an invaluable tool for the characterization of metallic nanoparticles, as it provides detailed information regarding their size, shape, and morphology. In the context of synthesizing AgNPs, TEM analysis can be utilized to evaluate external morphology, crystalline structure, and particle size [13]. For this purpose, the CM‐120 TEM instrument was employed, using carbon‐coated copper grids as a substrate for specimen preparation.

#### 2.4.5. FESEM

FESEM serves as a widely adopted method for evaluating the dimensions and structural features of NPs [14]. In this study, a Tescan instrument was utilized to examine the shape and particle size distribution of the synthesized AgNPs.

#### 2.4.6. EDX Spectroscopy

EDX spectroscopy was employed to investigate the elemental makeup of the synthesized NPs. This technique generated spectral data with distinct peaks that corresponded to particular elements within the electromagnetic emission range [13]. A Tescan instrument integrated with a scanning electron microscope was used to carry out the compositional analysis.

#### 2.4.7. XRD

XRD is a technique that helps to identify the crystalline structure, phase purity, space between planes, and degree of crystallinity of a given sample [12]. The relation between the distance of two planes (*d*) and the angle of diffraction (2*θ*) is given by Bragg’s equation (*nλ* = 2*d*sin *θ*), where *λ* is the wavelength of x rays and *n* is an integer known as the order of reflection (*h*, *k*, and *l* represent the Miller indices of the respective planes). The crystalline size was calculated using the Scherrer equation [12]. In this work, the XRD pattern of the synthesized AgNPs FEA@AgNPs was recorded using Cu‐Kα radiation (Å) in a 2*θ* range of 10°–80° under continuous scan mode with a step size of 0.05° and a counting time of 1 s per step.

### 2.5. Biological Activity Assays

#### 2.5.1. Cytotoxicity Assessment by MTT Assay

The cytotoxic effects of FEA@AgNPs and the crude FEA plant extract were evaluated in parallel on three cell lines: human gastric adenocarcinoma (AGS; IBRC C10071), PC3(C427), and HFFF2 cells—used as a normal control. AGS cells were provided by the IBRC, while PC3 and HFFF2 cells were obtained from the Pasteur Institute of Iran.

All cell lines were cultured under identical conditions in the RPMI‐1640 medium (Gibco) supplemented with 10% fetal bovine serum (FBS) and 1% penicillin–streptomycin and maintained at 37°C in a humidified atmosphere containing 5% CO_2_. Experiments were performed using cells from the third passage.

For the MTT assay, 1 × 10^4^ cells per well were seeded into 96‐well plates and allowed to adhere overnight. The medium was then replaced with fresh medium containing either FEA@AgNPs or the crude FEA extract at equivalent concentrations (20, 40, 80, 160, and 320 μg/mL, based on Ag or plant material content, respectively). Each treatment was performed in triplicate and applied simultaneously across all three cell lines.

After incubation for 24, 48, and 72 h, 20 μL of MTT solution (5 mg/mL in DPBS, filtered through a 0.2‐μm membrane) was added to each well, followed by 4 h of further incubation. The supernatant was removed, and formazan crystals were dissolved in 100 μL of DMSO. Absorbance was measured at 570 nm using a BioTek PowerWave XS2 microplate reader. Cell viability (%) was calculated relative to untreated controls using the following formula:

(1) percentage of viability=OD results of each sampleOD control×100.

##### 2.5.1.1. Statistical Analysis

The cytotoxicity assay was conducted with triplicate samples to ensure reliable results. Following the calculation of cell viability percentages using Microsoft Excel, the data were imported into SPSS Version 16 for statistical analysis. The experimental design followed a completely randomized model. Mean comparisons were carried out using Duncan’s multiple range test, and statistical significance was set at a *p* value of less than 0.05.

#### 2.5.2. Investigation of Antioxidant Properties Using the DPPH Assay

The DPPH assay is a widely used method for evaluating antioxidant capacity based on the ability of compounds to donate hydrogen atoms or electrons, thereby reducing the stable purple DPPH radical to its nonradical form. This reduction is accompanied by a measurable decrease in absorbance at 517 nm.

In this study, the antioxidant activities of green‐synthesized AgNPs (FEA@AgNPs), their corresponding plant extract, and ascorbic acid (as a positive control) were comparatively assessed. Solutions of FE@AgNPs and the plant extract were each prepared at concentrations of 20, 40, 80, and 160 μg/mL, while ascorbic acid was tested at equivalent concentrations for direct comparison. For each assay, 2 mL of a 100 μM methanolic DPPH solution was mixed with 2 mL of the respective sample, and the mixtures were incubated at room temperature for 30 min in the dark. Absorbance was then measured at 517 nm using a Spectramax Gemini XS spectrophotometer.

Antioxidant activity was expressed as percentage radical scavenging activity, calculated using the following formula:

(2) 100−sample absorbance−negative control sample absorbancenegative control sample absorbance×100,

where is the absorbance of DPPH with solvent only and corrects for any background absorbance from the sample itself. This experimental design enables a direct comparison between the antioxidant potential of the plant extract, the biosynthesized AgNPs, and the standard antioxidant ascorbic acid under identical conditions.

#### 2.5.3. Evaluation of Antimicrobial Properties of FEA@AgNPs and Aqueous Flower Extract of *E. Angustifolia*

The antimicrobial potential of plant‐mediated synthesized AgNPs, along with the corresponding plant extracts, was assessed using the agar well diffusion technique. This evaluation was performed against two bacterial strains—*S. aureus* (Gram‐positive) and *E. coli* (Gram‐negative)—as well as the fungal pathogen *C. albicans*.

Before testing, bacterial cultures were grown in nutrient broth at 37°C. Freshly prepared microbial suspensions, obtained from overnight cultures, were evenly spread onto agar plates using a sterile swab to achieve uniform coverage. Standardized wells were created in the agar and loaded with varying amounts of AgNP solutions and plant extract samples. The inoculated plates were then incubated at 37°C for 24 h. Following incubation, the formation of clear inhibition zones around the wells was visually examined, and their diameters were recorded. For antibacterial testing, gentamicin (20 μg per disk) served as the positive control. All plates were documented by digital photography. Each experiment was repeated three times, and the results are reported as mean ± standard deviation.

Also, the minimum inhibitory concentration (MIC) test was performed by diluting the sample concentrations in the microplate. In the presence of each dilution, bacteria were added to each well in an equal amount with a turbidity of about 0.1, and a well was considered a positive control without adding the substance, and another well was considered a sterility control without any bacteria. Then, the plate was incubated at 37°C for 24 h. Then, in the first dilution where the bacteria did not grow, the lowest concentration of the substance at which the bacteria did not grow was considered the MIC.

Then, to determine the minimum bactericidal concentration (MBC), these wells were cultured on the Mueller‐Hinton agar plate to see at which dilution the bacteria did not grow on the plate. MBC means the minimum concentration that leads to the death of the bacteria. This value may be different from the MIC.

### 2.6. Biomolecular Interaction Studies

#### 2.6.1. UV–Visible Spectroscopy for AgNPs Binding With HSA and DNA [15]

The interaction between ct‐DNA and AgNPs was investigated through UV–vis spectroscopic analysis using a NORDANTEC double‐beam spectrophotometer (T80 model). In these experiments, the ct‐DNA concentration was held constant at 55 μg/mL, while the FEA@AgNP concentration was varied within the range of 0.053–0.848 μg/mL. The resulting spectral changes were recorded in the wavelength range of 200–400 nm to monitor any shifts in DNA absorption characteristics upon binding to nanoparticles. All measurements were performed in a Tris–HCl buffer (0.1 M, pH 7.4) using a thermostated quartz cuvette maintained at 298 K.

In parallel, a stock solution of HSA was prepared by dissolving 0.0066 g of HSA in 2 mL of phosphate buffer (pH 7.4), yielding a final concentration of 5 × 10^−5^ M. This solution was stored at 4°C for 1 hour to ensure complete stabilization before use.

For the UV–vis titration of HSA with AgNPs, a fixed concentration of HSA (2.5 × 10^−6^ M) was exposed to increasing concentrations of AgNPs (0.053–0.795 μg/mL). Absorption spectra were again recorded in the range of 200–400 nm using the same spectrophotometer. The experimental procedures were conducted at 298 K using a thermostatically controlled quartz cuvette, with phosphate buffer (pH 7.4) serving as the reaction medium.

UV–vis spectra were recorded for HSA and ct‐DNA in their free forms as well as when bound to FEA@AgNPs, across a wavelength range of 200–400 nm. To calculate the apparent binding constants (*K*_app_K), variations in absorbance at 278 nm (for HSA) and 260 nm (for ct‐DNA) were analyzed following the nanoparticle interaction.

The following equation illustrates the equilibrium involved in the complexation process between HSA and AgNPs:

(3) HSA+AgNPs⟷KappHSA−AgNPs.

In this context, *K*_app_ refers to the apparent binding constant. The values of *K*_app_ were derived using the Benesi–Hildebrand (B–H) method, as described in the following:

(4) Aobs=1−αC0εHSA11+αC0εc.

In the above equation, *A*_obs_ represents the measured absorbance of the HSA solution in the presence of varying NP concentrations at 278 nm. The parameter *α* denotes the extent of binding between HSA and NPs. The molar absorptivity values for free HSA and the resulting HSA–NP complex at the same wavelength are indicated by *ε*_HSA_ and *ε*_*c*_, respectively. *C*_0_ corresponds to the initial concentration of HSA, while *l* refers to the path length of the cuvette used in the measurement.

By rearranging the terms of Equation (4), the expression can be transformed into the form presented in the following equation:

(5) Aobs=1−αA0+αAc.

In this context, *A*_0_ and *A*_*c*_ denote the absorbance values of free HSA and the HSA–NP complex, respectively, measured at 278 nm under the initial HSA concentration (*C*_0_). When the concentration of nanoparticles becomes sufficiently high, the association factor *α* can be approximated using the expression (*K*_app_ [NPs])/(1 + *K*_app_[NPs]), where [NPs] refers to the concentration of AgNPs.

Based on this approximation, Equation (5) can be reformulated into the form presented in

(6) 1Aobs−A0=1AC−A0+1KappAC−A0NPs.

Plotting 1/(*A*_obs_ − *i*_0_) against 1/[NPs] generates a B–H‐type curve. In this representation, the slope corresponds to 1/*K*_app_(*A*_*C*_ − *A*_0_), and the *y*‐intercept equals 1/(*A*_*C*_ − *A*_0_). The apparent binding constant (*K*_app_) can then be derived by taking the ratio of the intercept to the slope as defined in the above equation.

#### 2.6.2. Fluorescence Quenching Analysis [16]

Fluorescence spectra were recorded using a JASCO spectrofluorometer. In this set of experiments, the protein concentration was maintained at 2.50 × 10^−6^ M, while the concentration of AgNPs was varied between 0.053 and 0.848 μg/mL. Protein fluorescence emission was induced by excitation at 295 nm, with emission data collected over the range of 300–450 nm. Measurements were conducted at three distinct temperatures—288.15, 298.15, and 310.15 K—maintained using a circulating water system throughout the experimental runs.

It is well established that among the naturally occurring amino acids, phenylalanine (Phe), tryptophan (Trp), and tyrosine (Tyr) exhibit UV absorption due to their aromatic structures. To selectively monitor tryptophan fluorescence without interference from tyrosine or phenylalanine, an excitation wavelength of 295 nm was employed [16].

## 3. Results and Discussion

The present research focused on the eco‐friendly synthesis of AgNPs utilizing an aqueous extract derived from E. angustifolia flowers. The resulting nanoparticles were further evaluated for their antimicrobial activity, including antibacterial, antifungal, and antioxidant effects. To explore their biomedical applicability, the interaction behavior of AgNPs with DNA and HSA was analyzed employing UV–vis absorption and fluorescence spectroscopy. In addition, the cytotoxic potential of the synthesized AgNPs was examined against gastric cancer cells (AGS) and PC3 cell lines using the MTT assay.

### 3.1. Schematic Representation of Green Synthesis of AgNPs, Characterization, and Properties Investigation


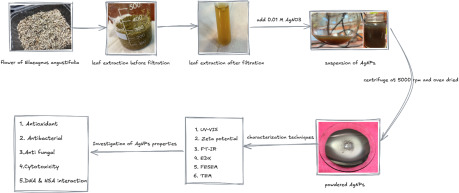


### 3.2. Phytochemical Screening of the Flower of *E. Angustifolia*

The use of plant‐derived compounds as reducing agents has emerged as a sustainable and environmentally friendly approach for the synthesis of AgNPs [17]. In recent years, various plant extracts have been investigated for their reducing properties and their potential applications in AgNP production [17]. A significant determinant in the synthesis of AgNPs is the nature of the reducing agent, as it directly affects features such as particle dimensions, structure, and long‐term stability [17]. Differences in the molecular structure of the reducing agent may strongly affect the physicochemical properties of the produced nanoparticles [17]. For instance, reducing agents that are abundant in phenolic or amine groups typically produce AgNPs with improved stability and biocompatibility [17].

The efficiency of a reducing agent—determined by its ability to donate electrons and reduce Ag^+^ ions—has a direct effect on the morphology and dimensions of the synthesized AgNPs. Agents with strong reducing power typically led to the formation of smaller particles, while those with lower activity tend to yield larger nanoparticles. Additionally, the reduction potential of the agent governs both the rate and completeness of the reduction reaction. A higher potential accelerates electron transfer, thereby enhancing the speed of nanoparticle formation [17].

Most phytochemicals extracted using polar solvents exhibit polar characteristics, and these compounds are known to actively participate in the biogenic synthesis of nanoparticles, often acting as both reducing and stabilizing agents [14].

Qualitative phytochemical analysis of the aqueous flower extract of *E. angustifolia* revealed the presence of five major bioactive compounds: phenols, alkaloids, flavonoids, terpenoids, and tannins. These phytochemicals serve dual roles in the synthesis of AgNPs, acting not only as reducing agents that convert Ag^+^ ions into nanoparticles but also as stabilizing and capping agents that inhibit particle aggregation and enhance colloidal stability. The complete results of the qualitative phytochemical screening are summarized in Table 2.

**TABLE 2:** Phytochemical analysis of the aqueous flower extract of Elaeagnus angustifolia.

No	Bioactive phytochemicals	Test/reagents	Result
1	FEA extraction	Blank	
2	Phenols	Ferric chloride test	+
3	Alkaloids	Mayer’s reagent	+
4	Flavonoids	Alkaline reagent test	+
5	Anthraquinones	Ferric chloride test	−
6	Terpenoids and steroids	Salkowski test	+ & −
7	Tannins	Ferric chloride test	+
8	Saponins	Frothing test	−

*Note:* Present: + and Absent: −.

### 3.3. Characterization of Synthesized AgNPs

To evaluate the formation, size, morphology, and structural features of the synthesized AgNPs, the following analytical techniques were utilized.

#### 3.3.1. UV–Vis Analysis [5]

UV–visible spectroscopy serves as a key analytical tool for monitoring the formation and optical properties of AgNPs. In this study, the biosynthesis of AgNPs was confirmed by the appearance of a SPR band in the range of 200–800 nm. A distinct absorption peak centered at 439 nm was observed (Figure 1(a)), characteristic of spherical AgNPs are attributed to the collective oscillation of conduction of electrons at the nanoparticle surface [2].

FIGURE 1(a) UV–vis absorption spectrum of FEA extraction, AgNPs and AgNO3 0.01 M. (b) Time‐dependent UV–vis spectra of the crude reaction mixture (unpurified) over 14 day: UV–vis absorption spectrum of a crude mixture of aqueous extraction of FEA and AgNP, after 2 h, after 7 days, and after 14 days of reaction time.

**Figure:**
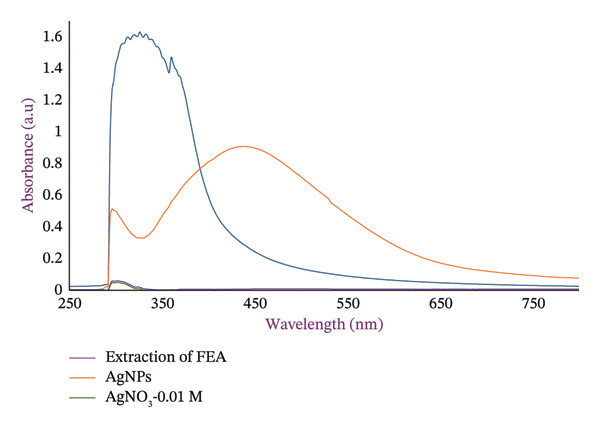
(a)

**Figure:**
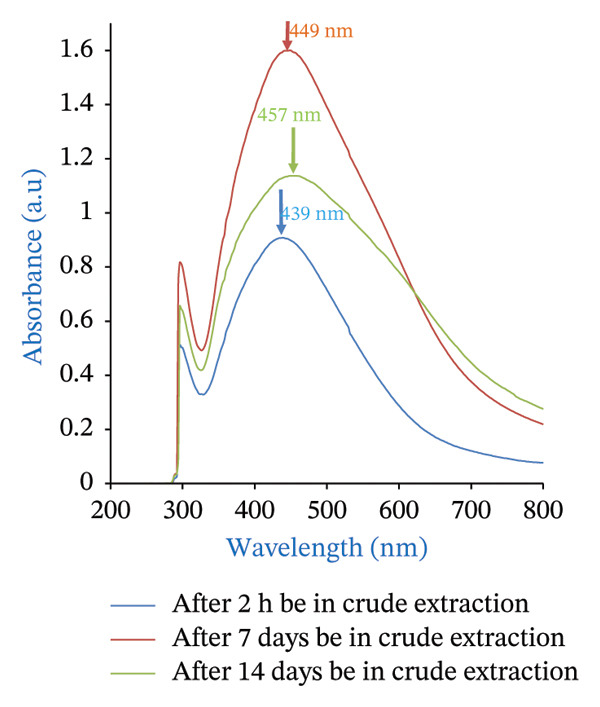
(b)

**FIGURE 2:**
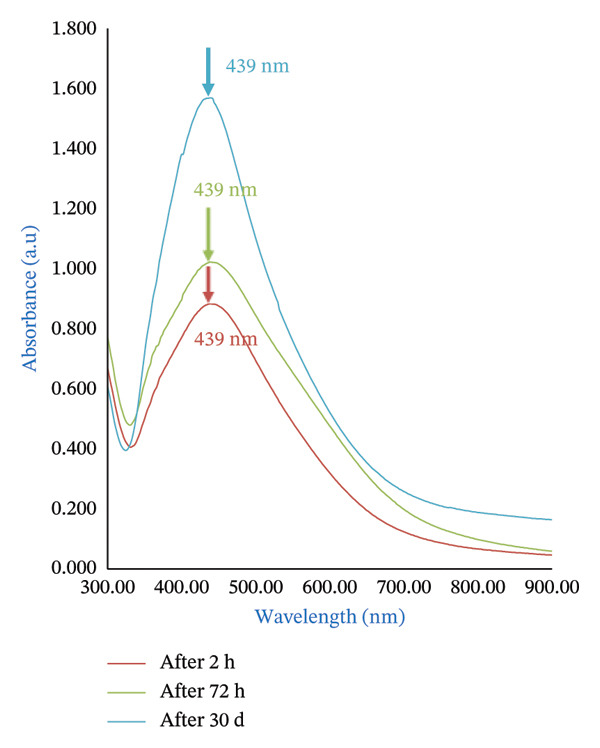
Long‐term stability of purified FEA@AgNPs in aqueous suspension over 30 days: UV–vis absorption spectrum of FEA‐AgNPs after 2 h, then centrifuged at 5000 rpm, and checked the UV–vis absorption spectrum after 72 h and 30 days.

To evaluate the kinetics and stability of the synthesis process, two sets of experiments were conducted.

i. Stability of the crude reaction mixture: The unpurified mixture of E. angustifolia floral extract and AgNO_3_ was monitored over 14 days (Figure 1(b)). A gradual red shift in the SPR peak—from 439 to 449 nm (Day 7) and 457 nm (Day 14)—was observed. This shift likely results from the ongoing reduction of residual Ag^+^ ions and/or incipient agglomeration in the presence of unreacted precursors and biomolecules, which is common in unpurified green synthesis systems.
ii. Stability of purified AgNPs: After optimizing synthesis conditions (2 h reaction time), the AgNPs were separated from the reaction medium by centrifugation, washed, and redispersed in DI water. The UV–vis spectrum of this purified colloid showed a stable SPR peak at 439 nm, with no significant shift observed even after 72 h and 30 days (Figure 2). This confirms the long‐term colloidal stability of the biosynthesized AgNPs, consistent with their high negative zeta potential (−32.68 mV), which prevents aggregation through electrostatic repulsion.

Together, these results indicate that while the crude reaction mixture exhibits time‐dependent changes due to residual reactants, the purified FEA@AgNPs are highly stable, making them suitable for downstream biomedical applications [18].

#### 3.3.2. Zeta Potential and DLS

The surface charge and colloidal stability of the biosynthesized FEA@AgNPs were evaluated by zeta potential analysis. As shown in Figure 3, the nanoparticles exhibited a highly negative zeta potential of −32.68 mV, indicating strong electrostatic repulsion between particles and confirming their excellent colloidal stability in aqueous suspension. The zeta potential distribution profile displayed a single, narrow, and symmetric peak centered at −32.68 mV, with FWHM of approximately 10–15 mV. This narrow distribution corroborates the formation of a relatively homogeneous nanoparticle population, a critical factor for reproducible biological performance [19, 20].

FIGURE 3Zeta potential of AgNPs synthesized with FEA ((a): after 2 h, (b): after 30 days).

**Figure:**
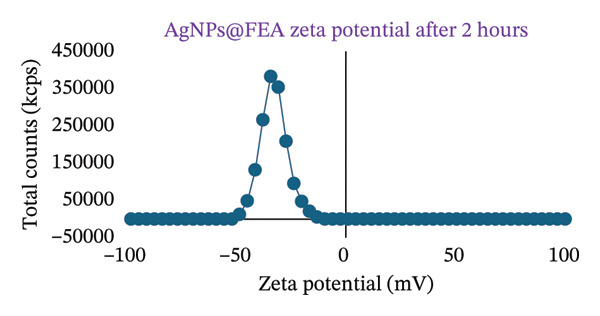
(a)

**Figure:**
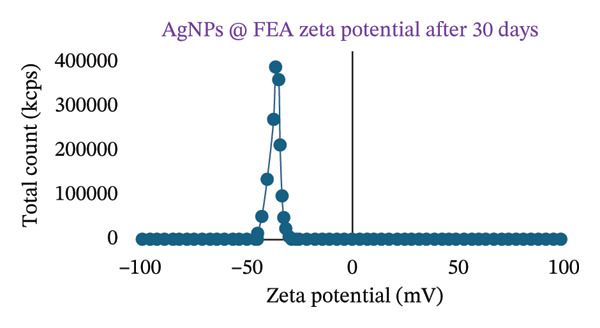
(b)

Furthermore, after 1 month of storage under ambient conditions, the nanoparticles retained excellent colloidal stability, exhibiting a zeta potential of −35.4 mV and a polydispersity index (PDI) of 0.19—values that continue to satisfy the criteria for long‐term stability and near monodispersity.

A zeta potential magnitude exceeding ±30 mV is widely accepted as a threshold for long‐term colloidal stability in aqueous media, as it ensures sufficient electrostatic repulsion to prevent aggregation [19, 20]. The observed negative surface charge likely arises from the adsorption of negatively charged phytochemicals (e.g., polyphenols, carboxylates, or flavonoids) from the flower of E. angustifolia extract onto the nanoparticle surface—a common feature in green‐synthesized AgNPs that also contributes to biocompatibility and steric stabilization [18, 19]. Complementary DLS analysis revealed a mean hydrodynamic diameter of 174 nm and a low PDI (0.18), indicating a relatively narrow size distribution. The significant discrepancy between the hydrodynamic size (174 nm) and the core particle dimensions observed by TEM (∼6.61 nm) and XRD (∼10 nm) is consistent with the presence of a thick phytochemical capping layer, hydration shell, and potential mild aggregation in solution—phenomena frequently reported for biogenic nanoparticles [18, 20, 21]. For instance, Khorrami et al. reported green‐synthesized AgNPs with a TEM size of ∼31 nm but a DLS hydrodynamic diameter of 51 nm, attributed to the protein corona formed by walnut extract [19]. Similarly, Oliveira et al. observed hydrodynamic sizes up to 9.7 nm (by TEM) vs. colloidal diameters exceeding 100 nm in DLS for Eucalyptus‐mediated AgNPs due to capping effects [18].

Notably, the PDI values of 0.18 and 0.19 fall well below the threshold of 0.2, which is considered indicative of near‐monodispersity in nanoparticle suspensions [20]. Combined with the high zeta potential (−32.68 mV) and (−35.4 mV), respectively, these parameters satisfy key criteria for colloidal stability and biocompatibility, as emphasized in recent reviews on nanomedicine design [20, 22]. While the large hydrodynamic diameter may limit systemic circulation, such particles remain well‐suited for topical applications, including antimicrobial coatings or localized anticancer therapy, where long‐term dispersion in storage and controlled biological interaction are essential [18, 19].

#### 3.3.3. FT‐IR Analysis

FT‐IR spectroscopy was employed to identify the biomolecules potentially responsible for the reduction of silver ions and the capping mechanism, which plays a crucial role in stabilizing the AgNPs synthesized using the extract of *E. angustifolia* flowers [23].

**FIGURE 4:**
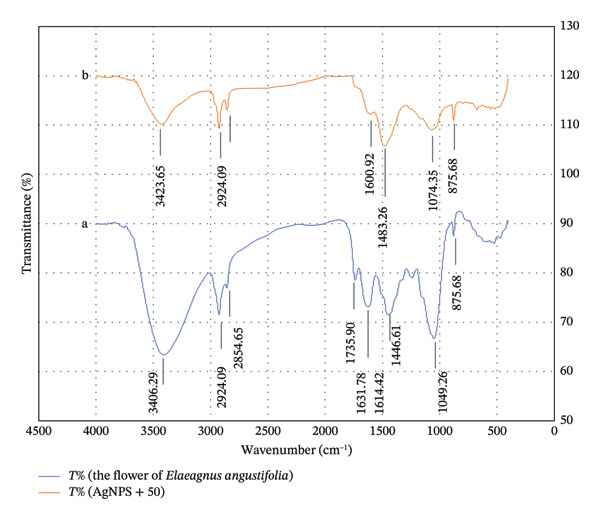
FT‐IR spectra of (a) Elaeagnus angustifolia flower extract (FEA) and (b) biosynthesized FEA‐AgNPs. Key functional groups involved in reduction and capping are labeled.

FT‐IR spectroscopy was conducted to investigate the functional groups that may play a role in the reduction, stabilization, and surface capping of AgNPs [23]. The spectral profile of the synthesized AgNPs was compared with that of the powder of flower buds without NPs. Both the biologically synthesized AgNPs and the *E. angustifolia* flower buds were scanned within the range of 400–4000 cm^−1^, as illustrated in Figure 4. This spectral comparison aids in identifying the possible biomolecules involved in the formation and stabilization of AgNPs generated using the E. angustifolia flower extract.

The FT‐IR spectra of the dried powder of *E. angustifolia* flower buds (FEA) and the biosynthesized AgNPs (FEA@AgNPs) are presented in Figures 4(a), 4(b), respectively.

##### 3.3.3.1. Figure 4(a) (FT‐IR of the Powder of the Flower of *E. Angustifolia*)

Figure 4(a) confirms the presence of various macromolecules based on the observed spectral peaks. The broad band at 3406 cm^−1^ corresponds to O–H stretching vibrations from alcohols, phenols, or carboxylic acids, as well as N–H stretching from amines or amides. Peaks at 2924 and 2854.65 cm^−1^ are attributed to asymmetric and symmetric C–H stretching vibrations in methyl groups. A distinct band at 1735.93 cm^−1^ indicates the presence of carbonyl (C=O) stretching vibrations, suggesting the existence of carboxylic acids, esters, ketones, or aldehydes—functional groups commonly found in flavonoids and tannins [24].

Additionally, bands at 1631.78 and 1614.42 cm^−1^ suggest C=C stretching or N–H bending vibrations. A peak at 1446.61 cm^−1^ may indicate the C–H bending modes [23].

In the fingerprint region, signals at 1240 and 1049 cm^−1^ correspond to O–H bending and C–O stretching vibrations of ester groups, respectively. These spectral features support the presence of phytochemicals such as phenols, flavonoids, and tannins, which are consistent with the results of earlier phytochemical tests. The prominent hydroxyl absorption bands further suggest the presence of phenolic compounds derived from the flower of *E. angustifolia* extract. Similar findings have been documented by Tesfye et al. [23].

##### 3.3.3.2. FT‐IR Spectra of the Synthesized AgNPs

Figure 4(b) illustrates the FT‐IR spectra of the biosynthesized AgNPs functionalized with biomolecules from *E. angustifolia* flowers, recorded in the spectral range of 400–4000 cm^−1^. Characteristic absorption bands were observed at 3423, 2924, 2852, 1735, 1600, 1483, 1074, 875, 673, and 553 cm^−1^, indicating the involvement of bioactive compounds in the capping and stabilization of FEA‐AgNPs [23].

The intense peak at 3423 cm^−1^ corresponds to O–H stretching vibrations, typically associated with phenolic and alcoholic groups, which are also present in the flower extract. The bands at 2924 and 2852 cm^−1^ are attributed to aldehydic C–H stretching vibrations, likely originating from flavonoid structures [25]. Peaks at 1735 cm^−1^ (C=O stretching of aldehydes, ketones, carboxylic acids, or esters), 1600 cm^−1^ (aromatic C=C), and 1483 cm^−1^ (C–H bending)—all of which are consistent with the presence of flavonoids, tannins, phenolic acids, and terpenoids [16, 24, 26]. A peak near 833 cm^−1^ (with a corresponding signal at 875 cm^−1^ in Figure 4(b)) was assigned to the C=CH_2_ functional group. Additionally, bands at 673 and 651.94 cm^−1^ are indicative of out‐of‐plane CH bending vibrations typical of cis‐substituted ethylene systems (–CH=CH–), further supporting the presence of organic molecules involved in NP stabilization [25].

The reduction mechanism is primarily attributed to the hydroxyl and carbonyl groups of these phytochemicals. Under aqueous conditions, phenolic –OH groups are oxidized to quinones or carbonyls, concomitantly donating electrons to reduce Ag^+^ to Ag^0^ [27]. This electron‐transfer process is facilitated by the high redox potential of polyphenols and flavonoids, which are abundant in *flowers of E. angustifolia*, as confirmed by prior phytochemical screening. The slight shift in the O–H stretching band from 3406 cm^−1^ (in the raw extract, Figure 4(a)) to 3423 cm^−1^ (in AgNPs, Figure 4(b)) suggests hydrogen bond disruption, which enhances the availability of phenolic protons for oxidation and supports their active role in Ag^+^ reduction [20].

Moreover, the stabilization (capping) of AgNPs is achieved through the coordination of oxygen‐ and nitrogen‐containing functional groups with the nanoparticle surface. The persistent presence of C=O (1735 cm^−1^), aromatic C=C (1600 cm^−1^), and C–O (1074 cm^−1^) vibrations in the AgNPs spectrum—alongside minor intensity changes compared to the raw extract—indicates that these biomolecules remain adsorbed onto the nanoparticle surface postsynthesis. This surface association creates a steric and electrostatic barrier that prevents aggregation and enhances colloidal stability, a mechanism widely reported in green‐synthesized AgNPs [26, 28]. Notably, capping is not merely a passive coating; it critically determines the biological efficacy and dispersion of AgNPs. As emphasized by Ansari et al. [29], stable, nonaggregated AgNPs exhibit superior antimicrobial and antibiofilm activity, as aggregation reduces the surface area and limits interaction with microbial membranes. In our system, the biomolecular capping derived from *E. angustifolia* flower buds ensures both nanoparticle stability and biofunctionality, aligning with established green synthesis principles.

In summary, *E. angustifolia* flower buds’ phytochemicals—particularly flavonoids and phenolics—reduce Ag^+^ via electron donation from hydroxyl/carbonyl groups and simultaneously cap the formed AgNPs through surface coordination. FT‐IR data directly corroborate this dual role by revealing the retention and slight modification of key functional groups after nanoparticle formation. This mechanism is consistent with the broader literature on plant‐mediated AgNP synthesis [26, 27, 30], confirming the green, efficient, and biocompatible nature of our synthesis approach.

#### 3.3.4. EDS [5]

**FIGURE 5:**
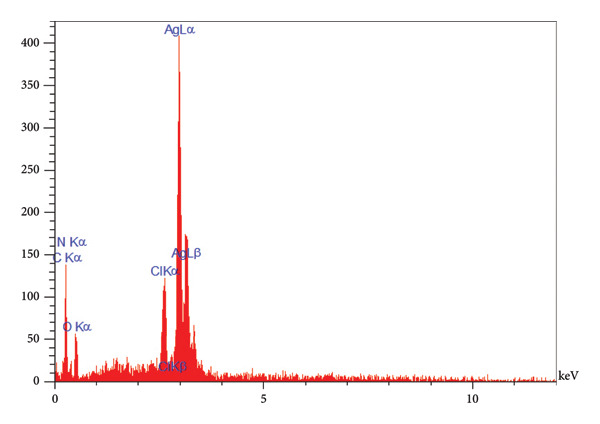
EDS spectrum of AgNPs from the flower of Elaeagnus angustifolia extract.

Figure 5 presents the EDS spectrum of AgNPs biosynthesized using an aqueous extract of *E. angustifolia* floral buds. A strong peak at approximately 3 keV corresponds to elemental silver, confirming the formation of AgNPs. Additional signals for carbon (C), oxygen (O), and nitrogen (N) are also observed, which are attributed to phytochemical capping agents derived from the plant extract adsorbed onto the nanoparticle surfaces [13].

The EDS analysis was performed using a Tescan scanning electron microscope equipped with an integrated EDS detector. Quantitative elemental composition is summarized in Table 3.

**TABLE 3:** Quantitative EDS analysis of biosynthesized AgNPs.

Element	Weight (%)	Atomic (%)	Likely origin
C	30.95	49.74	Organic capping layer (e.g., plant biomolecules)
N	13.57	18.70	Proteins, alkaloids, or other N‐containing phytochemicals
O	20.08	24.23	Hydroxyl/carboxyl groups in capping agents or adsorbed water
Cl	2.71	1.47	Residual Cl^−^ from the AgNO_3_ precursor or synthesis medium
Ag	32.69	5.85	Metallic silver core (Ag^0^/Ag^+^)
Total	100.00	100.00	

Silver constitutes the dominant metallic component (32.69 wt%), consistent with the expected composition of AgNPs. The presence of C, N, and O is the characteristic of biomolecular capping layers commonly observed in green‐synthesized nanoparticles.

A minor chlorine signal (2.71 wt%) was also detected, likely originating from residual chloride ions in the AgNO_3_ precursor or the synthesis medium. This concentration corresponds to approximately 3 mM NaCl—well within the low‐millimolar range reported in the literature to modulate nanoparticle growth and colloidal stability without impairing biological functionality [31]. In fact, trace Cl^−^ has been shown to act as a mild growth regulator, favoring the formation of smaller, well‐dispersed AgNPs (∼50 nm), which are often associated with enhanced antibacterial activity [31].

#### 3.3.5. FESEM [5]

**FIGURE 6:**
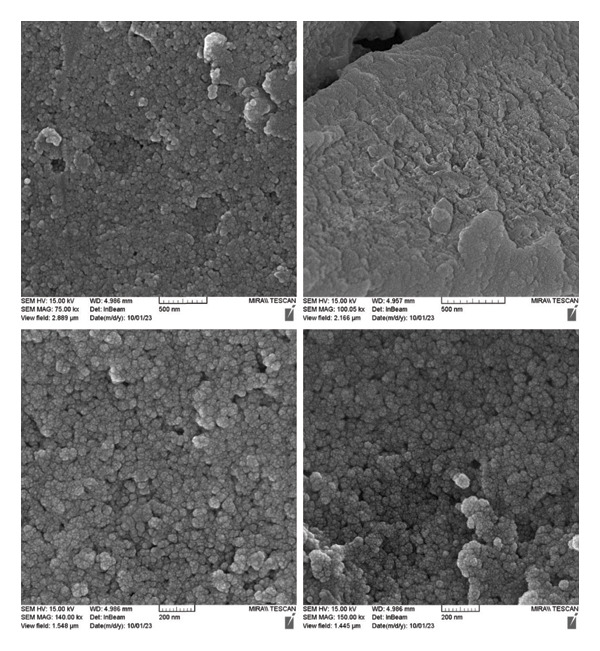
Surface morphology of synthesized silver nanoparticles from the aqueous extraction of flowers Elaeagnus angustifolia floral buds. Minor clustering is observed in FESEM images, likely due to drying artifacts during sample preparation, but no large aggregates are present, supporting colloidal stability as indicated by zeta potential (−32.68 mV) and DLS (PDI = 0.18).

FESEM was employed to evaluate the morphological features and particle size of the biosynthesized AgNPs, which were produced using the floral extract, operating at an accelerating voltage of 15.0 kV. The FESEM results, presented in Figure 6, reveal that the biosynthesized AgNPs exhibit a predominantly spherical morphology. These findings indicate that the extract from *E. angustifolia flowers* functions not only as a reducing agent but also as a stabilizing and capping agent during nanoparticle synthesis [5].

Also, the FESEM images clearly show the presence of aggregated structures. This aggregation is a common artifact observed during the drying process required for SEM sample preparation, where nanoparticles tend to cluster together as the solvent evaporates. It is important to note that this observed aggregation does not necessarily reflect the state of the nanoparticles in their native aqueous suspension.

To evaluate the true colloidal behavior and stability of the FEA@AgNPs in suspension, DLS and zeta potential analyses were performed. The DLS analysis revealed a hydrodynamic diameter of approximately 174 nm with a low PDI (0.18), indicating a relatively monodisperse population in solution. The measured hydrodynamic diameter is larger than the primary particle size estimated from high‐resolution imaging techniques like TEM (which typically measures the core particle size)—which is the average size of the AgNPs determined to be 6.61 nm, as DLS accounts for the entire hydrated particle, including the stabilizing capping layer derived from the *E. angustifolia* flower bud extract.

Crucially, the zeta potential analysis demonstrated a highly negative surface charge of −32.68 mV (Figure 3). A zeta potential magnitude exceeding ±30 mV is widely accepted as indicative of excellent colloidal stability due to strong electrostatic repulsion between particles [15]. This value is significantly more negative than that reported for AgNPs synthesized using Medicago sativa extract (−22.41 mV) [32], suggesting superior stability for our FEA@AgNPs. The narrow, symmetric peak in the zeta potential distribution (FWHM ≈ 10–15 mV) further confirms the homogeneity of the surface charge and the monodisperse nature of the suspension.

In conclusion, while FESEM imaging reveals aggregation artifacts inherent to the sample preparation method, the complementary DLS and zeta potential data provide compelling evidence that the FEA@AgNPs form a highly stable colloidal suspension in aqueous media. The strong negative surface charge, likely imparted by negatively charged biomolecules from the plant extract, which coated the nanoparticles [15], effectively prevents aggregation in solution through robust electrostatic repulsion, ensuring their suitability for subsequent applications.

#### 3.3.6. TEM

**FIGURE 7:**
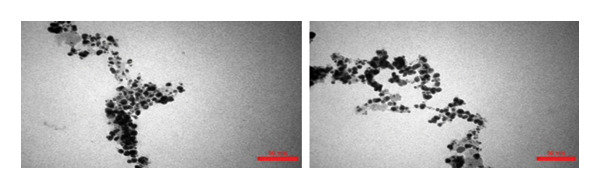
The morphology of silver nanoparticles synthesized using the extract of *Elaeagnus angustifolia* flowers via transmission electron microscopy (TEM).

TEM, a high‐resolution and sophisticated technique widely used for nanoparticle characterization and surface analysis, was employed to determine the primary particle size, morphology, and size distribution of the synthesized AgNPs in this study (Figure 7) [26]. The TEM images confirmed that the synthesized AgNPs exhibited a predominantly spherical morphology.

To quantitatively analyze the size distribution, the diameters of *n* = 55 individual nanoparticles were measured from multiple TEM micrographs. The measured particle sizes ranged from 1.71 to 12.06 nm. The statistical analysis of this dataset yielded the following key parameters.

**FIGURE 8:**
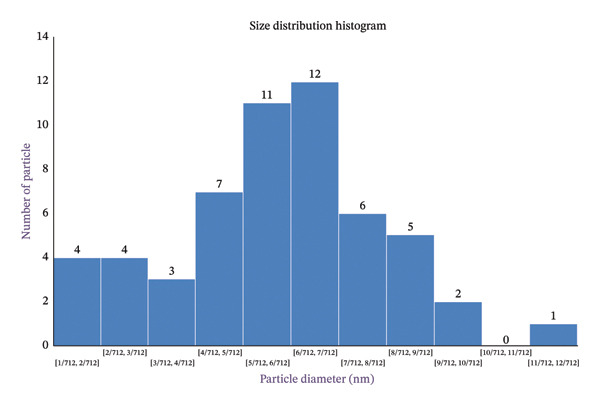
Size histogram.

 Mean diameter: 5.98 ± 2.18 nm, standard deviation (SD): 2.18 nm, median diameter: 6.61 nm
 Mode diameter: 7.03 nm (the most frequently occurring size within the measurement bins), range: 1.71–12.06 nm. These statistics are presented in the accompanying size distribution histogram (Figure 8).

The histogram (Figure 8) clearly illustrates the polydispersity of the sample, with a broad distribution centered around the mean value of 5.98 ± 2.18 nm and median value 6.61 nm. This primary particle size, as determined by TEM, is significantly smaller than the hydrodynamic diameter of 174 nm measured by DLS. This discrepancy is expected and commonly observed; DLS measures the hydrodynamic diameter, which includes the core nanoparticle plus its surrounding solvation layer and any stabilizing biomolecular capping agents derived from the *E. angustifolia* flower extract. In contrast, TEM measures the core physical diameter of the dried nanoparticle. The presence of aggregation visible in the FESEM images (Figure 6) further supports the notion that the DLS measurement reflects the size of particle clusters or agglomerates in suspension, while TEM provides the true size of the individual crystalline cores. The excellent colloidal stability demonstrated by the highly negative zeta potential (−32.68 mV) suggests that these aggregates observed in FESEM are primarily artifacts of the drying process for electron microscopy and not indicative of instability in the aqueous suspension.

In summary, TEM analysis confirms the successful synthesis of small, spherical AgNPs with a mean core diameter of 6.61 nm. The comparison between the TEM‐derived primary particle size and the DLS‐measured hydrodynamic diameter provides valuable insights into the structure of the nanoparticles in solution, highlighting the presence of a protective biomolecular corona and confirming the need to interpret different characterization techniques in context.

#### 3.3.7. X‐Ray Diffraction

**FIGURE 9:**
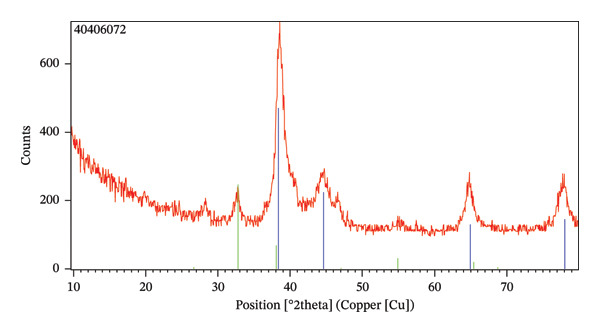
X‐ray diffraction (XRD) pattern of FEA@AgNPS.

XRD analysis of the green‐synthesized AgNPs confirmed the formation of a crystalline metallic phase with a face‐centered‐cubic (FCC) structure, consistent with the standard reference pattern for elemental silver (JCPDS No. 96‐901‐1609). The diffraction peaks observed at 2*θ* ≈ 38.1°, 44.1°, 64.5°, and 77.3° correspond to the (111), (200), (220), and (311) lattice planes, respectively—hallmarks of the FCC crystal system of metallic silver [4, 11, 33]. The calculated lattice parameter (*a* = 4.058 Å) and the high crystallinity of the sample closely align with previously reported values for pure Ag nanoparticles, providing strong evidence for the complete reduction of Ag^+^ ions to Ag^0^ during the plant‐mediated synthesis process [12, 33]. The corresponding XRD pattern of the FEA@AgNPs is shown in Figure 9.

In addition to the dominant Ag peaks, minor diffraction signals were detected that tentatively correspond to silver oxide (Ag_2_O, JCPDS No. 00‐041‐1104). However, these reflections exhibited notably low intensity (scale factor = 0.340 versus 0.650 for Ag), suggesting only trace or surface‐level oxidation. Given the inherent limitations of XRD in resolving low‐abundance secondary phases—and the potential for peak overlap between Ag and Ag_2_O—the presence of Ag_2_O cannot be definitively confirmed without complementary surface‐sensitive techniques such as x‐ray photoelectron spectroscopy (XPS) or HR‐TEM [4, 34]. This observation is consistent with prior reports on biogenic AgNPs, where minor oxide signatures are often attributed to postsynthetic surface oxidation upon atmospheric exposure [12, 35]. Notably, Jain et al. [33] underscored that plant‐derived capping agents—rich in polyphenols, flavonoids, and terpenoids—typically provide a protective layer that limits bulk oxidation, although superficial oxidation remains plausible under ambient conditions.

The average crystallite size was estimated using the Scherrer equation, based on the FWHM(0.840°) of the most intense (111) reflection at 2*θ* = 38.5°. This yielded a crystallite diameter of approximately 10 nm, a value well within the typical size range reported for green‐synthesized AgNPs. For instance, Dada et al. [4] reported AgNPs of 10–26 nm using the *Tithonia diversifolia* leaf extract, while Tesfaye et al. [12] observed even smaller crystallites (4.26–7.04 nm) with *Vernonia amygdalina*. Such nanoscale dimensions are highly favorable for biomedical applications, as smaller AgNPs exhibit enhanced antimicrobial and anticancer efficacy due to their high surface‐to‐volume ratio, improved cellular internalization, and greater ROS generation capacity [11, 12, 33].

Collectively, the XRD data corroborate the successful, predominantly pure synthesis of crystalline AgNPs via green chemistry. The structural and dimensional characteristics of the synthesized nanoparticles align closely with those reported in the recent literature, affirming the reliability of the phyto‐mediated approach. The tentative presence of a minor Ag_2_O phase—while not dominant—highlights the need for careful postsynthesis handling and storage (e.g., under an inert atmosphere or in dark, sealed containers) to preserve the structural integrity and functional efficacy of the nanoparticles, particularly in therapeutic contexts where redox stability is critical [33].

### 3.4. Phyto Nanoparticles for Antioxidant, Antibacterial, Antiviral, and Anticancer Activity and Biointeractions

The remarkably small size of the biosynthesized AgNPs (∼6.61 nm, as confirmed by TEM) is a critical determinant of their enhanced surface reactivity and multifunctional bioactivity. This ultrafine dimension places the particles firmly within the 1–10‐nm regime, where surface effects and quantum confinement dominate nanomaterial behavior, leading to amplified physicochemical and biological responses. The contribution of this nanoscale size to heightened reactivity can be explained through five interconnected mechanisms, all strongly corroborated by the current literature.

First, the surface area‐to‐volume ratio increases dramatically as particle size decreases below 10 nm. In ∼6.61 nm AgNPs, a substantial fraction of atoms (> 25%) resides on the surface, providing an abundance of reactive sites for interaction with biological targets—including microbial membranes, proteins, and nucleic acids. This geometric enhancement directly amplifies catalytic, redox, and binding capacities, a principle consistently observed in green‐synthesized AgNPs with sub‐10 nm dimensions exhibiting superior antimicrobial and antioxidant performance [32].

Second, ultrasmall AgNPs undergo more rapid oxidative dissolution in aqueous and physiological environments, leading to sustained and elevated release of Ag^+^ ions—the principal mediators of AgNP cytotoxicity. As emphasized by Marambio‐Jones & Hoek, the dissolution rate of AgNPs is proportional to their surface area; thus, nanoparticles under 10 nm release Ag^+^ more efficiently than larger counterparts, intensifying their biological impact [36].

Third, the diminutive size facilitates enhanced penetration across biological barriers. Kim et al. demonstrated that 10‐nm AgNPs induced significantly greater apoptosis in MC3T3‐E1 osteoblasts compared to 50‐ and 100‐nm particles, underscoring a clear size‐dependent cytotoxicity driven by improved cellular internalization and intracellular accumulation [37]. This enhanced membrane permeability extends to both eukaryotic and prokaryotic cells, enabling more effective targeting of intracellular components.

Fourth, high surface curvature and charge density in sub‐10 nm AgNPs promote stronger biomolecular interactions. Xu et al. noted that ultrasmall AgNPs exhibit heightened affinity for biomacromolecules such as DNA, attributed to their increased surface accessibility and electrostatic potential. This size‐dependent binding can induce conformational changes in DNA, disrupt replication, and amplify genotoxic and cytotoxic outcomes [38].

Finally, phytochemical capping in green‐synthesized AgNPs synergistically enhances size‐driven bioactivity. The bioactive corona—comprising phenolics, flavonoids, and other reducing phytoconstituents—does not merely stabilize the nanoparticle but actively modulates its biocompatibility, targeting, and redox behavior. As recently reviewed by Fahim et al. [17], sub‐10‐nm plant‐capped AgNPs can achieve a “dual functionality”: the metallic core drives Ag^+^–Ag‐mediated oxidative stress, while the phytochemical shell enhances selectivity toward cancer cells and reduces off‐target toxicity in normal cells [17].

#### 3.4.1. Antioxidant Activity

Antioxidants play a crucial role in protecting cells and tissues against oxidative stress induced by reactive free radicals, which can damage vital biomolecules such as DNA, proteins, and lipids, potentially leading to various pathological conditions [39]. AgNPs synthesized via green methods using plant extracts often exhibit notable antioxidant properties, attributed to both the intrinsic reactivity of the nanoparticles and the presence of bioactive phytochemicals involved in their synthesis [40]. Their small size (typically 1–10 nm) provides a high surface area‐to‐volume ratio, enhancing electron‐donating capacity and facilitating efficient neutralization of free radicals. Moreover, phytochemicals such as polyphenols, flavonoids, and tannins—acting as reducing and capping agents during synthesis—can remain associated with the nanoparticle surface, contributing synergistically to antioxidant effects [40].

In this study, the antioxidant potential of biosynthesized AgNPs, the aqueous floral extract of *E. angustifolia* (FEA), and ascorbic acid (as a positive control) was evaluated using the DPPH radical scavenging assay at concentrations of 20, 40, 80, and 160 μg/mL. The assay relies on the reduction of the purple DPPH radical to its yellow nonradical form upon interaction with antioxidants, with the degree of color change correlating to scavenging efficiency.

**FIGURE 10:**
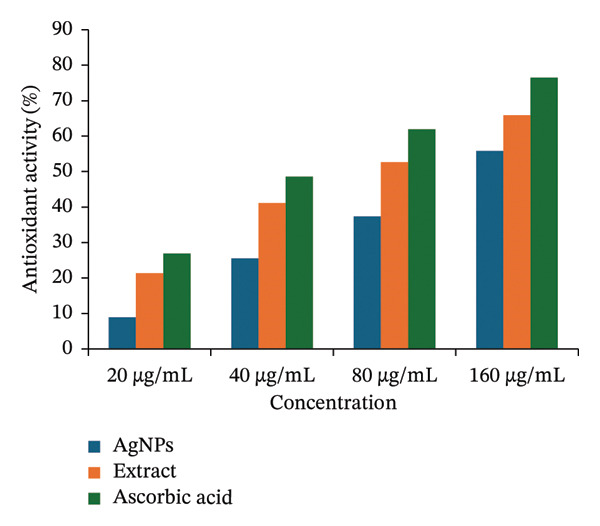
DPPH scavenging activity of different concentrations of AgNPs and extract compared to ascorbic acid.

As shown in Figure 10, all tested samples exhibited concentration‐dependent antioxidant activity. At the highest concentration (160 μg/mL), the FEA extract demonstrated the strongest activity (65% scavenging), followed closely by the AgNPs (55%), while ascorbic acid showed the highest efficacy (75%). The significantly higher activity of the crude extract compared to AgNPs (*p* < 0.05) is consistent with literature reports and may be attributed to the partial consumption or surface binding of antioxidant phytochemicals during nanoparticle synthesis. Although many bioactive compounds participate in the reduction and stabilization of Ag^+^ ions to form AgNPs, not all retain full free‐radical scavenging capacity in the final nanocomposite. Nevertheless, the considerable antioxidant activity retained by the AgNPs (55%) underscores the successful integration of functional phytochemicals onto the nanoparticle surface, supporting their potential utility in oxidative stress–related biomedical applications.

It is noteworthy that the observed reduction in the antioxidant capacity of AgNPs compared to the crude extract aligns with findings reported for *Thymus vulgaris* and *Allium sativum*, where green‐synthesized AgNPs exhibited lower DPPH scavenging activity (78% and 18%, respectively) than their parent extracts (85.9% and 62.7%) [40]. This contrasts with other systems—such as *Achillea millefolium* [41] or *Medicago sativa* [32]—where AgNPs showed enhanced antioxidant effects, likely due to synergistic interactions between the metallic core and residual phytochemicals. The variation underscores the dependency of antioxidant behavior on the specific phytochemical profile of the plant used. In our case, the moderate retention of activity (55%) suggests that while a fraction of redox‐active polyphenols was consumed during Ag^+^ reduction, a sufficient capping layer remained to confer notable radical scavenging potential.

#### 3.4.2. Antibacterial Activities [40]

The antibacterial efficacy of the *E. angustifolia* flower buds extract and green‐synthesized AgNPs was systematically evaluated against two representative bacterial strains: *E. coli* (Gram‐negative) and *S. aureus* (Gram‐positive), using well diffusion, MIC, and MBC assays.

##### 3.4.2.1. Antibacterial Activity of the Plant Extract

**FIGURE 11:**
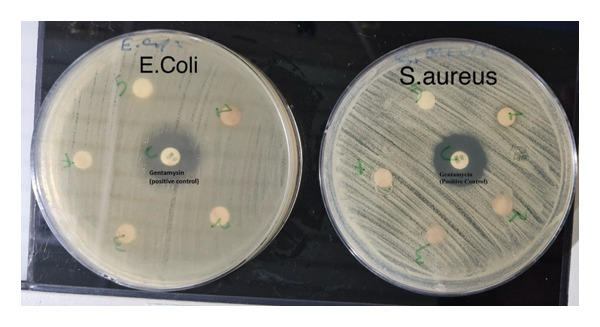
Zone of inhibition of the E. angustifolia flower buds extract at the various tested concentrations for *E. coli* and *S. aureus*, C: positive control (gentamycin).

The E. angustifolia flower buds extract was tested for its intrinsic antimicrobial activity via the well diffusion method. The results revealed no detectable zone of inhibition against either *E. coli* or *S. aureus* at the various tested concentrations. This indicates that, despite its richness in phytochemicals (as confirmed by FT‐IR), the extract alone lacks significant direct antibacterial activity under the experimental conditions. This is not uncommon, as many plant extracts exhibit biological activity only when their constituents are concentrated, purified, or transformed, such as through nanoparticle synthesis, as we describe more in the paragraphs below. Gentamicin (20 μg/disc) was used as a positive control and was placed at the center of each plate to serve as an internal reference for assay validity and to enable direct comparison of antimicrobial efficacy. The antimicrobial activity was assessed using the well diffusion method. A stock solution of the floral extract (5250 μg/mL) was prepared, and serial twofold dilutions were made to yield five concentrations: 5250, 2620.5, 1310.25, 650.6, and 320.8 μg/mL. Each dilution (100 μL) was loaded into 6‐mm wells punched in Mueller‐Hinton agar plates previously inoculated with a 0.5 McFarland bacterial suspension. Gentamicin (20 μg/disc) was placed at the center of each plate as a positive control. After incubation at 37°C for 24 h, no inhibition zones were detected for Gram‐negative and Gram‐positive bacterial strains. The representative results are presented in Figure 11.

##### 3.4.2.2. Antibacterial Activity of Green‐Synthesized AgNPs (FEA@AgNPs)

The antibacterial activity of biosynthesized AgNPs (FEA@AgNPs) was evaluated against *E. coli* (Gram‐negative) and *S. aureus* (Gram‐positive) using both broth microdilution for the quantitative determination of MIC and MBC, and agar well diffusion for the qualitative assessment of growth inhibition.

**FIGURE 12:**
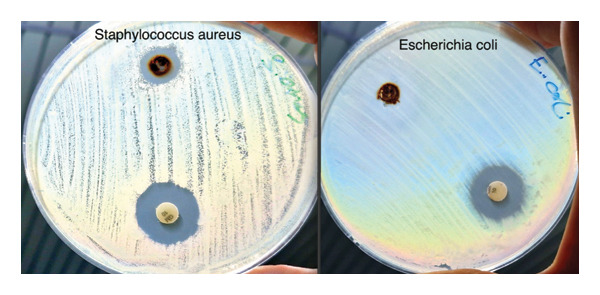
Antibacterial activities of (left) AgNPs against *S. aureus*, and (right) AgNPs against *E. coli* for 24 h (zone of inhibition of AgNPs‐EAF for *S. aureus* and *E. coli*, and positive control [gentamycin]).

The green‐synthesized AgNPs were evaluated to assess the role of nanoparticle formation in enhancing antimicrobial effects. In the well diffusion assay, no inhibition zone was observed against *E. coli*, indicating no significant activity against this Gram‐negative strain. The antimicrobial assay was repeated twice to confirm the effect of AgNPs on *E. coli*; however, no inhibition zone was detected in either trial. A 12‐mm inhibition zone was observed against *S. aureus*, while the inhibition zone of the positive control (gentamicin (20 μg/disc)) was 24 mm, demonstrating moderate activity against the Gram‐positive bacterium. The zone of inhibition patterns for FEA@AgNPs against *S. aureus* and *E. coli* is displayed in Figure 12.

**FIGURE 13:**
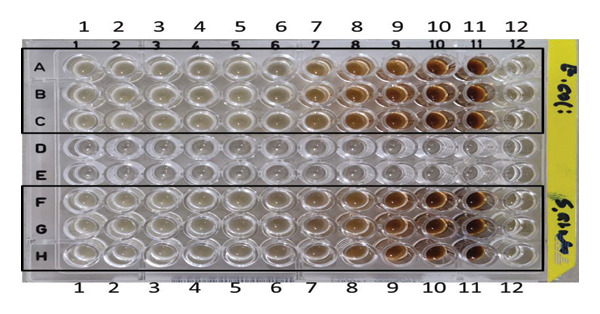
96‐well microtiter plate configuration for MIC testing, including sterility and growth controls for *E. coli* and *S. aureus*.

For quantitative assessment, the MIC and MBC were determined using the broth microdilution method in 96‐well microtiter plates according to standard protocols. Serial twofold dilutions of the green‐synthesized AgNPs were prepared in sterile broth, resulting in the following concentrations corresponding to wells 11 through 2: 600, 300, 150, 75, 37.5, 18.75, 9.37, 4.68, 2.34, and 1.17 (μg/mL). Well 12 served as the sterility control to confirm the absence of contamination in the culture medium, and Well 1 was considered a growth control. The configuration of the 96‐well microtiter plate, including sterility and growth controls for both bacterial strains, is schematically represented in Figure 13.

**FIGURE 14:**
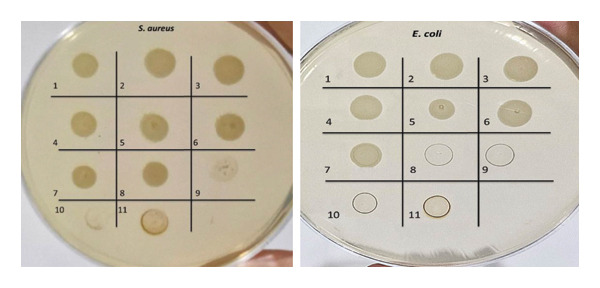
Representative subculture plate used in MBC assay: absence of visible bacterial growth confirms the bactericidal effect.

Each well was inoculated with 100 μL of bacterial suspension (final concentration ∼5 × 10^5^ CFU/mL) in MHB. The plates were incubated at 37°C for 24 h. The MIC was defined as the lowest concentration of FEA@AgNPs that completely inhibited visible bacterial growth. To determine the MBC, 100 μL of broth from wells showing no growth was subcultured onto fresh MHA plates and incubated for an additional 24 h; the MBC was recorded as the lowest concentration that resulted in ≥ 99.9% killing of the initial inoculum. A representative subculture plate confirming the absence of visible bacterial growth in MBC determination is presented in Figure 14.

The results revealed that FEA@AgNPs exhibited potent antibacterial activity against both tested strains. The MIC and MBC values for *E. coli* were 37.5 and 75 μg/mL, respectively, indicating bactericidal action at twice the MIC. In contrast, *S. aureus* showed higher resistance, with both MIC and MBC values of 150 μg/mL, suggesting that the nanoparticles exert a bactericidal effect at the same concentration required for growth inhibition. A comprehensive summary of the inhibition zone diameters, MIC, and MBC values for both bacterial strains is presented in Table 4.

**TABLE 4:** Summary of MIC and MBC values (in μg/mL) for the tested bacterial strains.

Strain	MIC (μg/mL)	MBC (μg/mL)	Inhibition zone (mm)
*E. coli*	37.5	75	No inhibition zone
*S. aureus*	150	150	12

The lower MIC and MBC values for *E. coli* compared to *S. aureus* suggest that AgNPs are more effective against the Gram‐negative strain in liquid culture, despite showing no diffusion zone in agar. This discrepancy may be due to the poor diffusion of nanoparticles through the agar matrix, aggregation of AgNPs in the solid medium, limiting mobility, or differences in cell wall interaction dynamics between liquid and solid environments.

The equal MIC and MBC for *S. aureus* indicate a bactericidal mode of action at the inhibitory concentration, likely due to AgNP‐induced membrane damage, protein denaturation, and ROS generation.

The enhanced antibacterial performance of FEA@AgNPs compared to the plant extract may be attributed to their smaller particle size, larger surface area, and the presence of bioactive molecules attached to their surface. Additionally, the antibacterial efficacy of AgNPs is influenced by factors such as the release of silver ions into the surrounding medium and the inherent susceptibility of the bacterial strains to these nanoparticles [27]. Smaller‐sized particles have shown more strong antibacterial activity [42] as they are more capable of entering bacteria. The smaller‐sized AgNPs exhibit a larger surface area than the larger ones, resulting in higher antibacterial activity [27].

The AgNPs (FEA@AgNPs) biosynthesized using aqueous extracts of *E. angustifolia* flower buds exhibited potent antibacterial activity, with MICs of 37.5 μg/mL against *E. coli* and 150 μg/mL against *S. aureus*. This marked enhancement in bioactivity compared to the crude extract aligns with a well‐established paradigm in green nanotechnology: Nanoparticle formation significantly amplifies the antimicrobial potential of plant metabolites. For example, Kambale et al. demonstrated that crude extracts of Congolese medicinal plants (*Brillantaisia patula*, *Crossopteryx febrifuga*, and *Senna siamea*) showed negligible activity (MIC > 10,000 μg/mL), whereas their AgNPs—especially those from *B. patula*—exhibited strong inhibition against human pathogens [43]. Similarly, Oliveira et al. reported that AgNPs synthesized from *E. grandis* leaf extract achieved an MIC of 53.9 μg/mL against *E. coli*, further corroborating that biogenic AgNPs can transform phytochemically rich but weakly active extracts into potent antimicrobial agents [18]. The consistently higher susceptibility of Gram‐negative *E. coli* relative to Gram‐positive *S. aureus* across these studies is likely due to structural differences in bacterial cell envelopes: While Gram‐negative bacteria possess an outer membrane that may facilitate nanoparticle interaction, the thick, cross‐linked peptidoglycan layer in Gram‐positive species acts as a permeability barrier that impedes AgNP penetration. Moreover, the comparable efficacy of silver nitrate at equivalent silver concentrations in related studies suggests that the sustained release of Ag^+^ ions through oxidative dissolution of the nanoparticles is a primary contributor to their bactericidal mechanism. Collectively, these findings underscore that FEA*@*AgNPs represent a promising candidate for therapeutic applications targeting Gram‐negative infections, particularly in an era of escalating antimicrobial resistance [18, 43].

It is noteworthy that while FEA@AgNPs exhibited no inhibition zone against *E. coli* in the agar well diffusion assay, they demonstrated potent bactericidal activity in broth microdilution, with an MIC of 37.5 μg/mL and an MBC of 75 μg/mL. This apparent discrepancy is well documented in the literature and is primarily attributed to the limited diffusion of AgNPs through the agar matrix, where particle aggregation and reduced mobility hinder their interaction with bacteria in solid media. In contrast, liquid culture (MIC/MBC assay) allows free nanoparticle dispersion and direct contact with bacterial cells, revealing their true antimicrobial potential. This phenomenon has been consistently observed in other studies: For example, Dada et al. [4] reported the antibacterial activity of *T. diversifolia*‐mediated AgNPs against *E. coli* only in MIC assays, with no diffusion zone in agar [4]; similarly, Al‐Fawwaz et al. observed negligible zones for *E. coli* despite confirmed AgNP efficacy [40].

Moreover, our MIC value of 37.5 μg/mL against *E. coli* is significantly lower than those reported for AgNPs derived from *Aconitum violaceum* (65 μg/mL) [39] and *Medicago sativa* (125 μg/mL) [32] and only marginally higher than the active walnut husk AgNPs (5–30 μg/mL) [19]. The higher resistance of *S. aureus* (MIC = MBC = 150 μg/mL), accompanied by a modest 12‐mm inhibition zone, aligns with trends from *Vernonia amygdalina* [12] and *Eucalyptus/T. arjuna* systems [44], likely due to the thick peptidoglycan layer in Gram‐positive bacteria that impedes nanoparticle penetration. Collectively, these comparisons confirm that FEA@AgNPs fall well within the expected spectrum of green‐synthesized antimicrobial AgNPs.

#### 3.4.3. Antifungal Activity of FEA@AgNPs [40]

**FIGURE 15:**
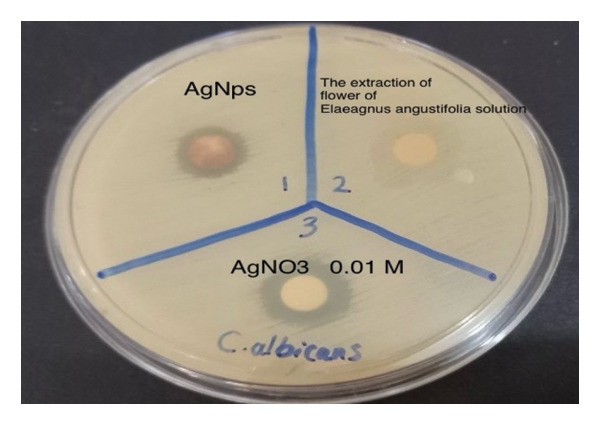
Antifungal activities of (1) FEA@AgNPs, (2) the extraction of the flower of Elaeagnus angustifolia solution, (3) AgNO_3_ 0.01 M against *Candida albicans*.

The antifungal potential of biosynthesized AgNPs (FEA@AgNPs), the aqueous floral extract of *E. angustifolia* (FEA), and silver nitrate (AgNO_3_, 0.01 M) was evaluated against *C. albicans* using the agar well diffusion assay (Figure 15). FEA@AgNPs exhibited a measurable inhibition zone of 12 mm, whereas no antifungal activity was observed for the crude FEA extract under identical experimental conditions. AgNO_3_ served as a positive ionic control and demonstrated slightly higher activity (14 mm), consistent with the known bioavailability of free Ag^+^ ions.

This pattern—inactivity of the plant extract alone coupled with significant antifungal efficacy of its AgNP derivative—is strongly corroborated by the recent literature. Alnahdi et al. reported that the aqueous root extract of Alkanna tinctoria showed zero inhibition against six Candida species (including *C. albicans*), while its AgNPs (At@AgNPs) produced inhibition zones ranging from 9.69 to 15.46 mm, with 11.29 mm against C. albicans—a result remarkably close to our 12 mm [45]. Similarly, Amini et al. observed that curcumin alone exhibited limited antifungal activity, but when formulated as curcumin‐coated AgNPs (Cur@AgNPs), it displayed MIC values significantly lower than fluconazole against C. albicans, underscoring the transformative role of nanoparticle synthesis in unlocking latent bioactivity [46].

In contrast, Al‐Fawwaz et al. documented a more nuanced relationship: for *Artemisia judaica* and *Thymus vulgaris*, AgNPs enhanced antifungal activity against C. albicans (28 and 25 mm, respectively) compared to their crude extracts (20 and 13 mm) [40]. However, for *Syzygium aromaticum*, *A. sativum*, and *Salvia rosmarinus*, the extracts outperformed their AgNPs [40]. This variability highlights that the biological outcome of green synthesis is highly dependent on the phytochemical profile of the source plant. In the case of *E. angustifolia* flower, our results align with Alnahdi et al. [45]: The extract is inactive, but the AgNPs are active—suggesting that the phytoconstituents (likely polyphenols and flavonoids) function primarily as reductants and capping agents during synthesis, and their integration onto the nanoparticle surface enables novel mechanisms of action not accessible in the free extract.

The antifungal mechanism of FEA@AgNPs likely involves a multimodal attack: (i) release of Ag^+^ ions disrupting cellular redox homeostasis; (ii) generation of ROS causing oxidative damage to lipids, proteins, and DNA; and (iii) physical interaction with the fungal cell membrane via the phytochemical corona, leading to increased permeability and cell death—mechanisms consistently reported across all three reference studies.

Thus, the emergence of antifungal activity only after nanoparticle formation is not an anomaly but a reproducible phenomenon in green nanobiotechnology. It reflects a fundamental principle: The biological identity of phytogenic nanoparticles is not the sum of plant extract and silver, but a new entity with emergent properties generated through bio–nano interactions.

#### 3.4.4. Anticancer Activity and Selective Cytotoxicity of FEA@AgNPs

To evaluate the anticancer potential and selectivity of *E. angustifolia* flower extract–mediated AgNPs (FEA@AgNPs), we assessed their cytotoxic effects on two human cancer cell lines—prostate carcinoma (PC3) and gastric adenocarcinoma (AGS)—alongside normal human dermal fibroblasts. Cell viability was measured using the MTT assay following exposure to FEA@AgNPs and the aqueous plant crude extract separately at concentrations of 20–320 μg/mL over 24, 48, and 72 h (Figures 16, 17, 18; Tables 5, 6, 7).

FIGURE 16Viability of cells (PC3‐cells) treated with different concentrations of FEA@AgNPs and *Elaeagnus angustifolia* crude extract for 24 (a), 48 (b), and 72 (c) hours, assessed by the MTT assay. Results are mean ± standard deviation (SD), and the letters from a to k indicate Duncan test; *p* < 0.05. *N* = three biological replicates for each treatment.

**Figure:**
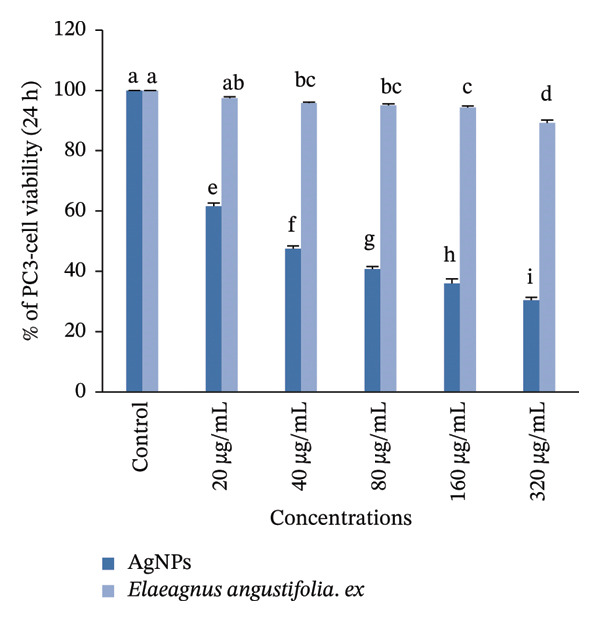
(a)

**Figure:**
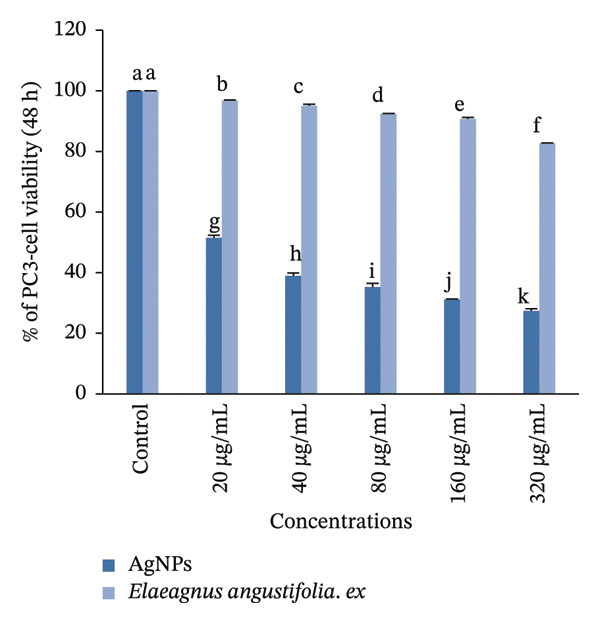
(b)

**Figure:**
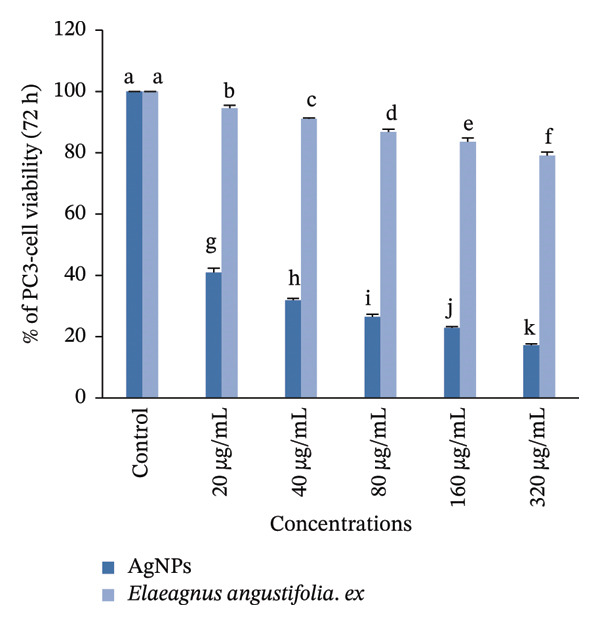
(c)

FIGURE 17Viability of cells (AGS cells) treated with different concentrations of FEA@AgNPs and *Elaeagnus angustifolia* flower bud crude extract for 24 (a), 48 (b), and 72 (c) hours, assessed by the MTT assay. The results are mean ± standard deviation (SD), and the letters from a to k indicate Duncan test; *p* < 0.05. *N* = three biological replicates for each treatment.

**Figure:**
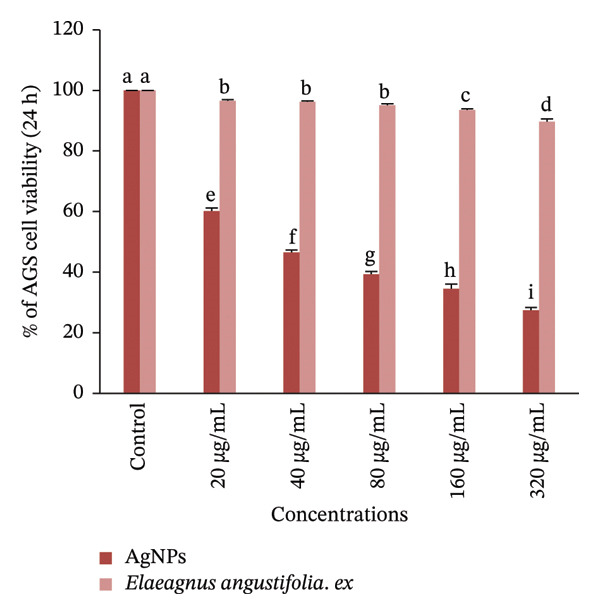
(a)

**Figure:**
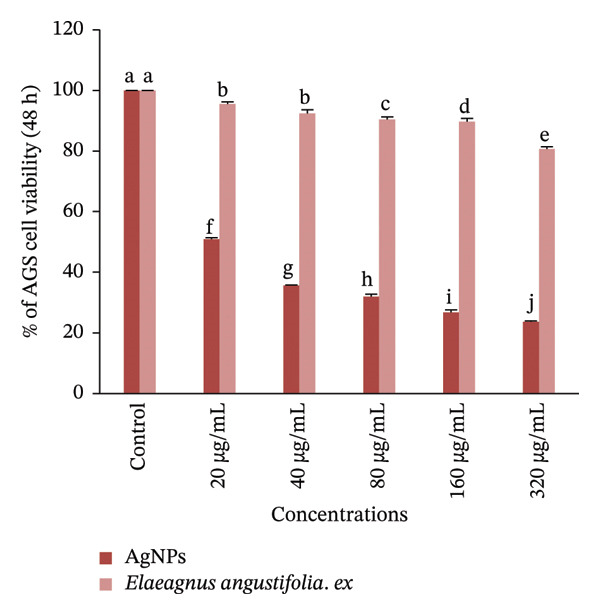
(b)

**Figure:**
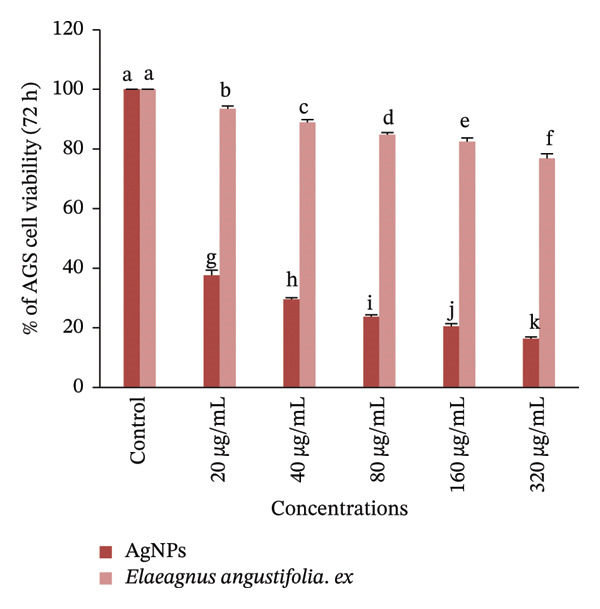
(c)

FIGURE 18Viability of cells (fibroblast cells) treated with different concentrations of FEA@AgNPs and *Elaeagnus angustifolia* crude extract for 24 (a), 48 (b), and 72 (c) hours assessed by the MTT assay. The results are mean ± standard deviation (SD), and the letters a to k indicate Duncan test; *p* < 0.05. *N* = three biological replicates for each treatment.

**Figure:**
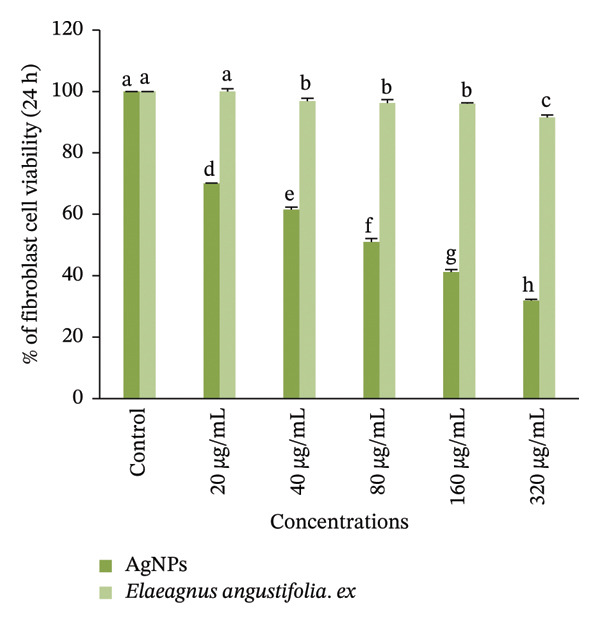
(a)

**Figure:**
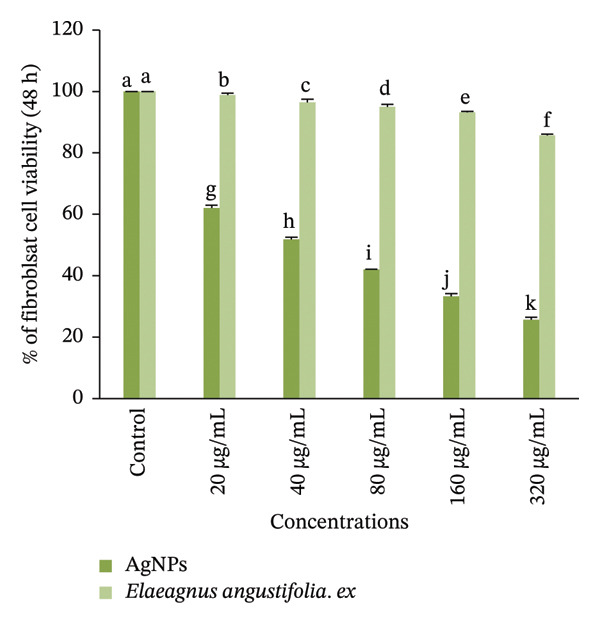
(b)

**Figure:**
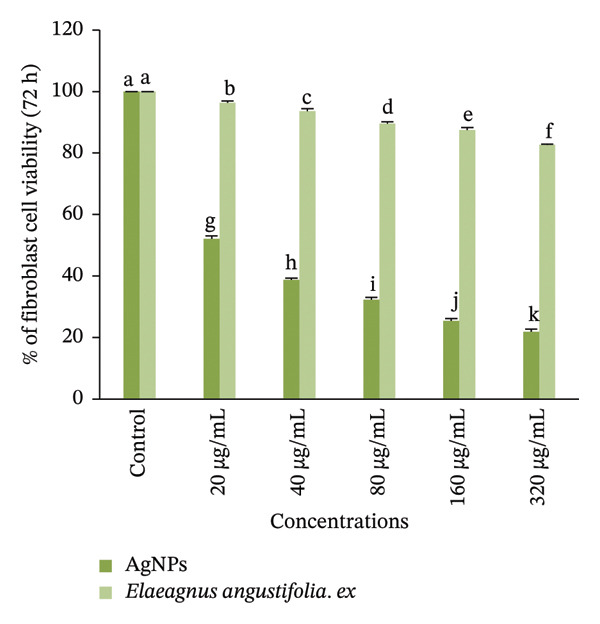
(c)

**TABLE 5:** Viability of cells (PC3 cells) treated with different concentrations of FEA@AgNPs and *Elaeagnus angustifolia* flower bud aqueous extract for 24, 48, and 72 h, assessed by the MTT assay.

Treatment	24 h	48 h	72 h
Control	100^a^	100^a^	100^a^
20 μg/mL of AgNPs	61.57 ± 2.43^e^	51.45 ± 0.88^g^	40.88 ± 1.41^g^
40 μg/mL of AgNPs	47.55 ± 2.21^f^	38.94 ± 0.95^h^	31.81 ± 0.63^h^
80 μg/mL of AgNPs	40.73 ± 2.03^g^	35.27 ± 1.20^i^	26.39 ± 0.82^i^
160 μg/mL of AgNPs	35.96 ± 1.12^h^	31.14 ± 0.13^j^	22.84 ± 0.38^j^
320 μg/mL of AgNPs	30.41 ± 1.51^i^	27.33 ± 0.77^k^	17.21 ± 0.42^k^
20 μg/mL of *Elaeagnus angustifolia* extract	97.40 ± 0.87^ab^	96.72 ± 0.24^b^	96.32 ± 0.98^b^
40 μg/mL of *Elaeagnus angustifolia* extract	95.73 ± 1.48^bc^	95.00 ± 0.55^c^	93.62 ± 0.28^c^
80 μg/mL of *Elaeagnus angustifolia* extract	95.02 ± 0.95^bc^	92.40 ± 0.16^d^	89.48 ± 0.95^d^
160 μg/mL of *Elaeagnus angustifolia* extract	94.36 ± 0.70^c^	90.64 ± 0.49^e^	87.43 ± 1.22^e^
320 μg/mL of *Elaeagnus angustifolia* extract	89.26 ± 1.77^d^	82.68 ± 0.09^f^	82.64 ± 1.14^f^

*Note:* Results are shown as mean ± SD (*n* = 3). Different superscripts within the same column demonstrate significant differences (*p* < 0.05), whereas the same superscripts do not demonstrate significant differences (*p* > 0.05).

**TABLE 6:** Viability of cells (AGS) treated with different concentrations of FEA@AgNPs and Elaeagnus angustifolia flower extract for 24, 48, and 72 h assessed by the MTT assay.

Treatments	24 h	48 h	72 h
Control g	100^a^	100^a^	100^a^
20 μg/mL of AgNPs	60.13 ± 1.03^e^	50.94 ± 0.52^f^	37.67 ± 1.67^g^
40 μg/mL of AgNPs	46.44 ± 0.91^f^	35.66 ± 0.15^g^	29.62 ± 0.44^h^
80 μg/mL of AgNPs	39.31 ± 0.86^g^	31.94 ± 0.85^h^	23.70 ± 0.70^i^
160 μg/mL of AgNPs	34.48 ± 1.60^h^	26.87 ± 0.77^i^	20.43 ± 0.99^j^
320 μg/mL of AgNPs	27.37 ± 0.97^i^	23.69 ± 0.27^j^	16.32 ± 0.68^k^
20 μg/mL of *Elaeagnus angustifolia* extract	96.46 ± 0.42^b^	95.53 ± 0.74^b^	93.45 ± 0.93^b^
40 μg/mL of *Elaeagnus angustifolia* extract	96.20 ± 0.31^b^	92.33 ± 1.33^b^	88.96 ± 0.90^c^
80 μg/mL of *Elaeagnus angustifolia* extract	95.01 ± 0.53^b^	90.37 ± 0.89^c^	84.74 ± 0.83^d^
160 μg/mL of *Elaeagnus angustifolia* extract	93.46 ± 0.47^c^	89.66 ± 1.02^d^	82.42 ± 1.25^e^
320 μg/mL of *Elaeagnus angustifolia* extract	89.66 ± 0.89^d^	80.71 ± 0.64^e^	76.83 ± 1.62^f^

*Note:* Different superscripts within the same column demonstrate the significant differences (*p* < 0.05), whereas the same superscripts do not demonstrate significant differences (*p* > 0.05). The results are shown as mean ± SD (*n* = 3).

**TABLE 7:** Viability of cells (fibroblast cells) treated with different concentrations of FEA@ AgNPs and Elaeagnus angustifolia flower crude extract for 24, 48, and 72 h assessed by the MTT assay.

Treatments	24 h	48 h	72 h
Control g	100^a^	100^a^	100^a^
20 μg/mL of AgNPs	70.06 ± 0.10^d^	61.96 ± 0.98^g^	52.14 ± 0.93^g^
40 μg/mL of AgNPs	61.48 ± 0.84^e^	51.76 ± 0.78^h^	38.80 ± 0.50^h^
80 μg/mL of AgNPs	50.95 ± 1.16^f^	42.04 ± 0.05^i^	32.35 ± 0.67^i^
160 μg/mL of AgNPs	41.19 ± 0.75^g^	33.29 ± 0.92^j^	25.47 ± 0.72^j^
320 μg/mL of AgNPs	31.91 ± 0.41^h^	25.63 ± 0.78^k^	21.88 ± 0.82^k^
20 μg/mL of *Elaeagnus angustifolia* extract	99.91 ± 0.94^a^	98.78 ± 0.67^b^	96.32 ± 0.55^b^
40 μg/mL of *Elaeagnus angustifolia* extract	96.82 ± 0.89^b^	96.35 ± 1.04^c^	93.62 ± 0.78^c^
80 μg/mL of *Elaeagnus angustifolia* extract	96.24 ± 1.10^b^	94.98 ± 0.78^d^	89.84 ± 0.62^d^
160 μg/mL of *Elaeagnus angustifolia* extract	95.98 ± 0.37^b^	93.15 ± 0.33^e^	87.43 ± 0.83^e^
320 μg/mL of *Elaeagnus angustifolia* extract	91.51 ± 0.86^c^	85.70 ± 0.38^f^	82.64 ± 0.16^f^

*Note:* Different superscripts within the same column demonstrate the significant differences (*p* < 0.05), whereas the same superscripts do not demonstrate significant differences (*p* > 0.05). The results are shown as mean ± SD (*n* = 3).

Dose‐ and time‐dependent cytotoxicity was observed for FEA@AgNPs across all cell lines, with significantly higher potency against cancer cells compared to normal fibroblasts (*p* < 0.05). In contrast, the plant extract alone exhibited markedly lower cytotoxicity, even at the highest tested concentration (320 μg/mL), consistent with its traditional use as a safe herbal remedy.

To quantify therapeutic selectivity, the half‐maximal inhibitory concentration (IC_50_) values were calculated and are summarized in Table 8. FEA@AgNPs demonstrated potent activity against AGS (IC_50_ = 5.33 μg/mL) and PC3 (IC_50_ = 7.49 μg/mL) cells after 72 h, while requiring a substantially higher concentration to achieve comparable toxicity in fibroblasts (IC_50_ = 19.79 μg/mL). This corresponds to selectivity indices (SI = IC_50_, fibroblast/IC_50_, cancer) of ∼3.7 for AGS and ∼2.6 for PC3, indicating a preferential targeting of malignant cells.

**TABLE 8:** IC_50_ values (μg/mL) of FEA@AgNPs and E. angustifolia aqueous extract against human cancer and normal cell lines after 24, 48, and 72 h of treatment.

Cell line	Component	Time interval (h)	IC50 (μg/mL)
PC3	FEA@AgNPS	24	42.13
PC3	FEA@AgNPS	48	16.93
PC3	FEA@AgNPS	72	7.49
AGS	FEA@AgNPS	24	35.48
AGS	FEA@AgNPS	48	15.58
AGS	FEA@AgNPS	72	5.33
Fibroblast	FEA@AgNPS	24	86.91
Fibroblast	FEA@AgNPS	48	46.30
Fibroblast	FEA@AgNPS	72	19.79
PC3	Extraction	24	18424
PC3	Extraction	48	3378
PC3	Extraction	72	5452
AGS	Extraction	24	31102
AGS	Extraction	48	4804
AGS	Extraction	72	4901
Fibroblast	Extraction	24	9363
Fibroblast	Extraction	48	2711
Fibroblast	Extraction	72	5526

Finally, IC_50_ values exceeding the highest tested concentration (320 μg/mL), with values of 4901 μg/mL (AGS), 5452 μg/mL (PC3), and 5526 μg/mL (fibroblasts) after 72 h, further support the safety of the phytochemical matrix.

The selective cytotoxicity of FEA@AgNPs—evidenced by IC_50_ values of 5.33 μg/mL (AGS) and 7.49 μg/mL (PC3) versus 19.79 μg/mL in normal fibroblasts—aligns with the emerging paradigm that green‐synthesized AgNPs can function as “two‐in‐one” therapeutic platforms, combining intrinsic silver‐driven oxidative stress with phytochemical‐mediated targeting [47]. This selectivity is not unique to *E. angustifolia* flower buds; similar patterns have been reported for walnut husk–derived AgNPs, which induced markedly higher cytotoxicity in MCF‐7 breast cancer cells than in L‐929 fibroblasts (70% vs. 15% cell death), attributed to ROS‐mediated apoptosis and selective transcriptional inhibition [19]. Moreover, the biocompatibility of plant‐capped AgNPs extends beyond fibroblasts: Mussin et al. observed no toxicity in human peripheral blood mononuclear cells (PBMCs) at concentrations up to 16 μg/mL, suggesting that the phytochemical corona mitigates off‐target effects even in an immunologically sensitive context [48]. Notably, our IC_50_ value in normal fibroblasts (19.79 μg/mL) closely matches that reported by Zare‐Bidaki et al. for *Medicago sativa*–derived AgNPs (IC_50_ = 18.22 μg/mL), further supporting the biocompatibility of plant‐capped AgNPs in normal human cells [32].

Recent reviews further corroborate this trend, noting that green‐synthesized AgNPs (FEA@AgNps) consistently demonstrate preferential toxicity toward malignant cells while sparing noncancerous counterparts—a selectivity rarely observed with chemically synthesized analogs [49]. In stark contrast, chemically synthesized AgNPs—such as citrate‐capped variants—exhibit indiscriminate cytotoxicity, with an IC_50_ as low as 2.06 μg/mL reported in normal Vero kidney cells [50]. This dichotomy underscores the critical role of the phytochemical corona in modulating bio–nano interactions [47]. As emphasized by Kovács et al., the therapeutic window of AgNPs is largely determined not by inherent silver ion sensitivity, but by cellular uptake efficiency—a process heavily influenced by nanoparticle size and surface chemistry [47]. The ultrasmall size of FEA@AgNPs (6.61 nm) likely enhances internalization in metabolically active carcinoma cells, while the polyphenol‐rich capping derived from *E. angustifolia* flower buds may attenuate unintended interactions with normal fibroblasts. This dual advantage—enhanced tumor cell penetration combined with biocompatible surface functionality—aligns with the proposed mechanism whereby green‐synthesized AgNPs induce apoptosis through ROS‐mediated mitochondrial dysfunction, DNA damage, and caspase activation, preferentially in malignant cells [33, 47].

Collectively, these attributes position FEA@AgNPs as a rational candidate for localized anticancer strategies—particularly in postsurgical or topical applications, where sustained, site‐specific activity with minimal systemic exposure is therapeutically advantageous [47].

#### 3.4.5. Interaction of FEA@AgNPs With HSA and ct‐DNA: UV–Vis Spectroscopic Analysis

UV–visible absorption spectroscopy serves as a sensitive and noninvasive tool to probe the binding interactions between nanomaterials and key biological macromolecules such as HSA and double‐stranded DNA. To evaluate the binding affinity of green‐synthesized FEA@AgNPs toward HSA and ct‐DNA, titration experiments were conducted at 298 K under physiological conditions (Figure 19).

FIGURE 19UV–visible absorption spectra of (a) human serum albumin (HSA) and (b) calf thymus DNA (ct‐DNA) in the absence and presence of increasing concentrations of silver nanoparticles (AgNPs). (a) Absorption profiles of HSA (1.7 × 10^−5^ M) alone and with escalating AgNP concentrations (0.27–1.94 μM). (b) Absorption spectra of Ct‐DNA (55 μg/mL) alone and in the presence of increasing AgNP concentrations (0–80 μg/mL).

**Figure:**
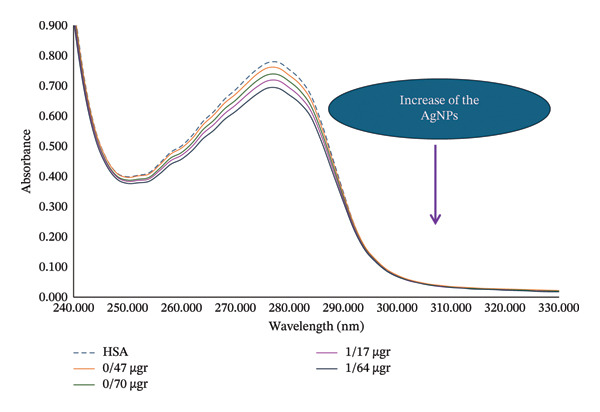
(a)

**Figure:**
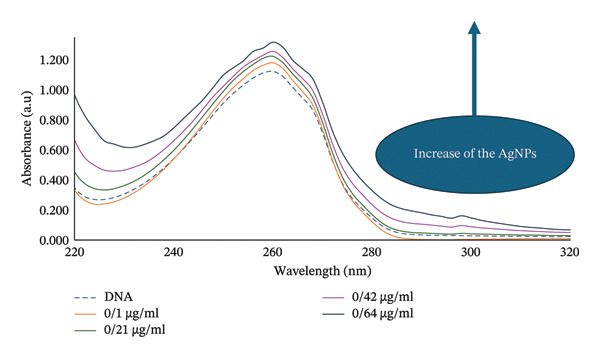
(b)

##### 3.4.5.1. Interaction With HSA

HSA (1.7 × 10^−5^ M) exhibits a characteristic absorption band at 279 nm, attributed to *π* ⟶ *π*∗ transitions in the aromatic residues (tryptophan, tyrosine, and phenylalanine). Upon incremental addition of FEA@AgNPs, a gradual hypochromic effect was observed at 279 nm without any significant shift in λ_max_ (Figure 19(a), inset). This spectral behavior indicates that FEA@AgNPs interact with HSA primarily through surface adsorption, rather than penetrating the hydrophobic core or significantly perturbing the local microenvironment of the aromatic residues. Such adsorption likely leads to the rapid formation of a protein corona, comprising a dynamic “soft corona” and a tightly bound “hard corona” [51, 52]. Using the B–H method ((6), the binding constant (*K*_*b*_) for the FEA@AgNPs–HSA complex was determined to be 6.6 × 10^−2^ M^−1^—a value consistent with moderate‐to‐strong protein–nanoparticle interactions reported in the literature [53].

##### 3.4.5.2. Interaction With ct‐DNA

The interaction of FEA@AgNPs with ct‐DNA (55 μg/mL) was monitored by tracking absorbance changes at 260 nm—the signature peak for DNA base stacking. Upon titration with increasing AgNP concentrations (0–80 μg/mL), a clear hyperchromic effect emerged without any shift in the absorption maximum (Figure 19(b)). This increase in absorbance suggests partial unwinding of the DNA double helix, resulting in greater exposure of nitrogenous bases— a hallmark of intercalative or groove‐binding interactions [54]. The apparent binding constant (*K*b) for the FEA@AgNPs–ct‐DNA complex was calculated as 5.38 × 10^6^ M^−1^, indicating a significantly stronger affinity for DNA than for HSA.

This preferential binding may be attributed to electrostatic attraction between the positively charged surface of FEA@AgNPs (as inferred from zeta potential measurements in related studies) and the negatively charged phosphate backbone of DNA. Moreover, the observed hyperchromicity aligns well with recent reports on AgNP–DNA interactions, including the study by Ali et al. [15] and the comprehensive biophysical analysis by Shahabadi and Mahdavi [54], who identified a partial intercalation mode involving both electrostatic and stacking forces.

Collectively, the UV–vis data reveal that FEA@AgNPs bind to both HSA and ct‐DNA, albeit through distinct mechanisms and with markedly different affinities. While HSA binding suggests the formation of a biocompatible corona with minimal structural perturbation, the strong DNA interaction—characteristic of genotoxic potential—warrants further investigation into the biological safety of these nanoparticles in therapeutic applications.

##### 3.4.5.3. Mechanism of Binding Mode (Stern–Volmer Analysis)

In this experiment, we employed the Stern–Volmer quenching equation [16] to analyze the fluorescence quenching data and to deliberate the quenching mechanism of AgNPs with HSA.

(7) F0F=11+Kqτ0Q=+KsvQ.

In the mentioned equation, *F*_0_ and *F* are the emission intensities of albumin without and with AgNPs, respectively.

*K*_sv_ is the Stern–Volmer collisional quenching constant, *K*_*q*_ is the quenching rate constant of albumin, [*Q*] is the concentration of AgNPs, and *τ*_0_ demonstrates the molecular average lifetime of albumin without collisional (about 10 nSec) [16].

**FIGURE 20:**
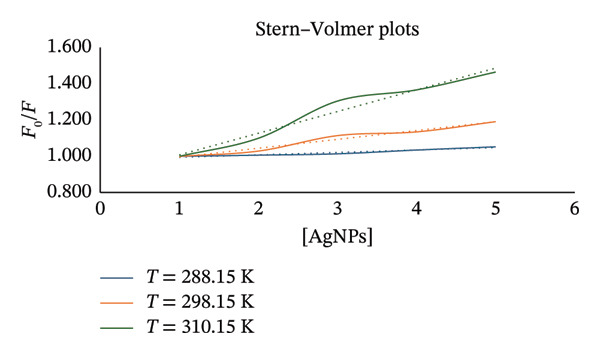
Stern–Volmer plots for the binding of the AgNPs to HSA at 288.15, 298.15, and 310.15 K.

As can be seen in Figures 19 and 20, the Stern–Volmer quenching constant (*K*_sv_) increases with temperature. This behavior is characteristic of dynamic quenching, where the rate of collisions between the fluorophore (HSA tryptophan) and the quencher (AgNPs) increases with temperature [16] (Table 9).

**TABLE 9:** Binding parameters of the interaction of AgNPs to HAS.

Temperature	*K*_sv_ (mL/μg)	*R*_2_ value
288.15 K^0^	1.33 × 10^−2^	0.9432
298.15 K^0^	4.88 × 10^−2^	0.9695
310.15 K^0^	11.97 × 10^−2^	0.9687

In conclusion, the strong binding affinity of FEA@AgNPs toward ct‐DNA (*K*_*b*_ = 5.38 × 10^6^ M^−1^), coupled with hyperchromicity without peak shift, suggests partial unwinding of the DNA duplex—likely due to the electrostatic interaction between positively charged AgNPs and the phosphate backbone, or groove binding as reported by Thomas et al. [55]. This structural perturbation may interfere with replication and transcription, a mechanism that underpins both the anticancer potential (as seen in our IC_50_ = 5.33–7.49 μg/mL against AGS/PC3) and the risk of genotoxicity at higher doses [19].

Conversely, the moderate binding to HSA (*K*_*b*_ = 66 × 10^−3^ M^−1^) with no spectral shift indicates surface adsorption without denaturation of the protein’s secondary structure. Computational studies confirm that such binding typically occurs in the hydrophobic drug‐binding pocket of Subdomain IIA [51], which is known to prolong systemic circulation and enhance biodistribution. Furthermore, Li et al. demonstrated that HSA‐dominated protein coronas stabilize AgNPs in physiological media, preventing aggregation and reducing rapid clearance [52].

Thus, while DNA binding may drive therapeutic (anticancer) or adverse (genotoxic) outcomes depending on the context, HSA interaction likely improves pharmacokinetic performance and biocompatibility—a dual advantage that supports the potential of FEA@AgNPs for targeted biomedical applications.

## 4. Conclusion

In summary, AgNPs biosynthesized using *E. angustifolia* flower extract (FEA@AgNPs) demonstrated potent antibacterial activity against *E. coli* and *S. aureus*, and selective cytotoxicity toward AGS and PC3 cancer cell lines, with significantly lower toxicity in human fibroblasts. However, their antifungal and antioxidant effects were only moderate, and the study’s novelty stems largely from the use of an underexplored botanical source rather than a transformative methodological or mechanistic advance. While preliminary biocompatibility data in fibroblasts are encouraging, more comprehensive cytotoxicity profiling across diverse normal human cell types—particularly under conditions paralleling anticancer assays—remains necessary to substantiate claims of selectivity.

Given these constraints, the most plausible near‐term applications for FEA@AgNPs lie in localized or topical settings, such as antimicrobial wound dressings, catheter coatings, or dermal formulations for accessible lesions, where systemic exposure is minimized.

Future studies should prioritize

i. evaluation against multidrug‐resistant clinical isolates (e.g., MRSA, ESBL‐producing Enterobacteriaceae);
ii. in vivo toxicology, biodistribution, and pharmacokinetic assessments; and
iii. combinatorial studies with standard therapeutic agents to investigate potential synergistic effects that could enhance efficacy while reducing required doses.

## Funding

No funding was received for this manuscript.

## Conflicts of Interest

The authors declare no conflicts of interest.

## Data Availability

The data that support the findings of this study are available on request from the corresponding author. The data are not publicly available due to privacy or ethical restrictions.
